# Effect of Throat Cross-Sectional Aspect Ratio on the Cavitation Characteristics of Flat-Slot Cavitating Water-Jet Nozzles: A Flow-Field Design Basis for Flake Graphite Exfoliation

**DOI:** 10.3390/ma19194129

**Published:** 2026-09-27

**Authors:** Yun Jiang, Xing Dong, Dongsheng Li, Jiaxing Li, Lu Chang, Chenhao Guo

**Affiliations:** 1 School of Safety and Emergency Engineering, Heilongjiang University of Science and Technology, Harbin 150022, China; jiangyun011010@163.com; 2 School of Mechanical Engineering, Heilongjiang University of Science and Technology, Harbin 150022, China18145974766@163.com (C.G.)

**Keywords:** cavitating water jet, flat-slot nozzle, aspect ratio, numerical simulation, cavitation characteristics

## Abstract

Flat-slot nozzles with noncircular throat cross-sections have potential applications in liquid-phase exfoliation of flake graphite. This study investigated the effect of throat aspect ratio (AR) on cavitation using the Mixture multiphase, SST *k*-*ω* turbulence, and Zwart–Gerber–Belamri cavitation models for one circular nozzle (AR = 1) and four flat-slot nozzles (AR = 3, 5, 7, and 9). High-speed imaging compared macroscopic cavitation-cloud morphologies and their variations. At a constant throat cross-sectional area, relative differences in peak time-averaged centerline velocity were below 1%, indicating limited AR effects on peak jet velocity. However, AR markedly altered the near-exit velocity field and cavitation-cloud distribution. Increasing AR enhanced lateral spreading of the high-speed core and accelerated downstream centerline velocity decay. The global maximum of the time-averaged vapor volume fraction field changed only slightly, decreasing initially and then increasing. For flat-slot nozzles, peak time-averaged centerline vapor volume fraction increased markedly from 39.26% (AR = 3) to 77.84% (AR = 9), a 98.27% relative increase. Cavitation progressively extended from localized near-wall regions toward the jet center. High-speed imaging showed cavitation-cloud morphologies and AR trends generally consistent with simulations. These findings clarify AR-induced cavitation redistribution and provide guidance for nozzle design in future flake graphite exfoliation studies.

## 1. Introduction

Cavitation is a phase-change phenomenon in which a liquid vaporizes as a result of a local pressure reduction [1,2,3]. Previous studies have shown that the substantial energy released during the rapid collapse of cavitation bubbles can generate microjets directed toward nearby solid surfaces, thereby imposing intense mechanical loading on those surfaces [4]. Cavitating water-jet technology exploits this mechanism by deliberately inducing cavitation within a jet and using the impact generated by bubble collapse to enhance its erosive capability [5]. Consequently, this technology has been widely applied to cleaning and descaling [6,7], material surface strengthening [8,9], and microforming [10].

The nozzle is a key component of a water-jet system. Its geometry and structural parameters not only determine the cavitation intensity of the cavitating jet and the stability of cavitation-cloud development [11,12,13] but also affect the interaction of the jet with the material [14]. In recent years, extensive numerical and experimental studies have been conducted to investigate the relationship between nozzle structure and cavitation performance. Using transient numerical simulations, Dong et al. [15] revealed a nonmonotonic effect of the chamber-wall arc radius on cavitation performance. When the radius was 2 mm, the cavitation area increased by 122.5% and the turbulent kinetic energy increased by 16.9% compared with those of the chamber with piecewise-linear walls. Through numerical simulations and experiments, Shi et al. [16] found that a double-cavity self-excited oscillation nozzle exhibited more favorable pressure-pulsation characteristics than a conventional organ-pipe nozzle and substantially increased the cleaning pressure on the target surface. Using CFD simulations and erosion experiments, Wu et al. [17] reported that the erosion efficiency of an organ-pipe nozzle was optimized when the divergence angle was 15°, the throat length was equal to one throat diameter, and the diverging-section length was equal to three throat diameters. Numerical results obtained by Fang et al. [18] using dynamic mode decomposition (DMD) showed that the Helmholtz nozzle produced the highest jet energy with quasi-periodic oscillations, the organ-pipe nozzle generated the highest peak axial velocity and maintained stable coherent structures, and the Venturi nozzle provided the greatest jet dispersion and cavitation performance. Using flow-visualization experiments and large-eddy simulation (LES), Liu et al. [19] demonstrated that a cylindrical nozzle outperformed organ-pipe and converging-diverging nozzles in terms of both cavitation-cloud size and vapor volume fraction. Using VOF-LES parametric simulations, He et al. [20] numerically found that cavitation intensity in the throat was positively correlated with the convergence angle and that optimizing the convergence and divergence angles could effectively balance cavitation performance and energy consumption. Through ceramic-tile erosion tests, Zhang et al. [21] reported that gradually converging-abruptly expanding and gradually converging-gradually expanding nozzles exhibited substantially greater cavitation-erosion capability than conventional nozzles. These studies demonstrate that the axial structural parameters of conventional cavitating nozzles can substantially affect pressure fluctuations, flow separation, cavitation development, and jet erosion performance, thereby providing an important basis for nozzle structural optimization.

In addition to conventional axisymmetric nozzles, the effects of noncircular cross-sections and their aspect ratios on jet structure have also received considerable attention. In studies of noncavitating jets, AR has been treated as an independent geometric variable and investigated through multilevel parametric analyses. Zhao et al. [22] combined high-speed imaging experiments with three-dimensional VOF-LES numerical simulations to investigate water-jet morphologies produced by elliptical and diamond-shaped nozzles with three ARs. They divided the evolution of noncircular water jets into four stages— incomplete axis switching, complete axis switching, jet instability, and breakup—and reported that AR and curvature affected the number of axis-switching events by altering the two-component vorticity distribution. Bhide et al. [23] used steady RANS simulations based on the SST *k*-*ω* model to systematically compare the flow characteristics of rectangular nozzles with five ARs. They found that rectangular nozzles with higher ARs weakened shock waves, reduced boundary-layer thickness, and delayed jet axis switching. Teramoto et al. [24] used constant-temperature hot-wire anemometry to measure the mean and fluctuating velocities of multiple elliptic jet arrays and investigated the effects of two ARs and different circumferential arrangements on jet development. They found that, with an alternating arrangement of nozzles having a higher AR, the jet at the center of the array did not undergo axis switching. These studies demonstrate that, even in the absence of cavitation, AR itself can substantially affect the spatial structure of noncircular jets by altering cross-sectional confinement, shear-layer development, and vortex-structure evolution.

Studies of cavitating flows involving the AR of noncircular flow passages have generally treated a specific flat-slot or elliptical cross-section as a single geometric configuration and compared it with a circular nozzle, or focused on the effects of orifice orientation and turbulence models. Yin et al. [25] used a VOF-Spray one-way coupling model to compare the internal flow and external spray characteristics of elliptical and circular nozzles and found that the cavitation regions and turbulent disturbances of the AR = 4 elliptical nozzle were mainly concentrated along the major-axis direction. Hong et al. [26] used transparent acrylic nozzles and a CCD camera to visualize the internal cavitating flow and external liquid jet and showed that cavitation occurred in an elliptical nozzle at a lower Reynolds number than in a circular nozzle and that the overall spray angle of the elliptical nozzle was larger. Wang et al. [27] used detached-eddy simulation (DES) combined with the Zwart–Gerber–Belamri cavitation model to investigate the effect of different orientations of elliptical diesel orifices and found that orifice orientation markedly affected cavitation inception, potential cavitation-erosion regions, and exit turbulence intensity. Wu et al. [28] used a CFD method based on the Schnerr–Sauer cavitation model to compare flat-type, conventional, and Y-shaped nozzles and found that a flat-type nozzle produced a larger vapor volume fraction and a wider axial cavitation distribution than conventional and Y-shaped nozzles. In addition, Apte et al. [29] conducted two- and three-dimensional numerical simulations of a rectangular Venturi cavitation channel, compared URANS and DES-type turbulence modeling approaches, and validated the simulations using high-fidelity experimental data. Their results showed that flow dimensionality and turbulence modeling approach markedly affected the prediction of unsteady cavitation morphology and cavitation-turbulence interactions. Therefore, the existing literature clearly demonstrates that cavitating flows in noncircular cross-sections have already been investigated and that cross-sectional shape, orientation, and three-dimensional turbulence effects can all substantially influence cavitation development.

However, in terms of the variables considered in the above studies, although existing studies of noncircular cavitating nozzles have employed numerical simulations, visualization experiments, and turbulence modeling approaches at different scales to reveal the effects of cross-sectional shape and orifice orientation on cavitation development, AR has generally been treated as an inherent parameter of a specific noncircular geometric configuration rather than being systematically investigated at multiple levels as an independent variable while maintaining the same throat cross-sectional area and principal axial geometric parameters. In particular, for racetrack-shaped flat-slot cavitating nozzles, increasing AR is accompanied by a reduction in cross-sectional height and hydraulic diameter, an increase in wetted perimeter, and changes in wall confinement along different directions. How these geometric changes collectively affect the internal and external velocity fields, the distributions of cavitating regions in different central planes, and the axial development of cavitation in the jet-center region has not yet been systematically compared. Therefore, a systematic investigation combining numerical simulations and visualization experiments is required to characterize the internal and external flow fields and cavitation-cloud distributions under different AR conditions.

During the water-jet exfoliation of flake graphite, microjet impact and frictional shear are the primary mechanisms promoting lamellar separation [30]. At an identical throat cross-sectional area, a flat-slot nozzle produces a substantially greater velocity gradient in the height, or minor-axis, direction of the throat cross-section than a circular nozzle. This characteristic can intensify frictional shear within the jet and may therefore facilitate preferential exfoliation along the graphite interlayers. Meanwhile, the redistribution of cavitating regions induced by variations in AR may further alter the spatial locations of cavitation-bubble-collapse effects within the jet cross-section. Based on these flow characteristics, systematically investigating the effects of AR on the velocity field and cavitation distribution of flat-slot nozzles can provide a flow-field basis for screening candidate nozzle parameters in subsequent flake graphite exfoliation studies.

To address the issues identified above, the throat cross-sectional aspect ratio was treated as the primary geometric variable in this study. Numerical simulations were conducted in ANSYS Fluent 2024 R2 for one circular reference nozzle and four flat-slot nozzles with different ARs while maintaining the same throat cross-sectional area and principal axial geometric parameters. The simulations were used to compare the velocity fields and vapor volume fraction distributions, while cavitating water-jet visualization experiments were performed to examine the corresponding macroscopic cavitation-cloud morphologies. The objectives were to distinguish the influence of AR on peak jet velocity from its influence on the spatial structure of the velocity field and to investigate the effects of AR on cavitation intensity, the spatial distribution of cavitating regions, and cavitation development in the jet-center region. In contrast to previous studies focused mainly on performance comparisons among different cross-sectional shapes, the present work examined multiple AR levels to systematically characterize the evolution of the cavitating flow field as the flat-slot throat cross-section became progressively flatter. High-speed imaging was further used to provide qualitative, trend-level validation of the numerically predicted macroscopic cavitation-cloud morphologies and their AR-dependent variations. Notably, this study focused on nozzle flow-field characteristics. The resulting velocity-field and cavitation-cloud distributions are intended primarily to support the selection of candidate nozzle parameters for subsequent flake graphite exfoliation experiments and to provide flow-field-level guidance for nozzle design.

## 2. Numerical Models and Experimental Methods

### 2.1. Flat-Slot Cavitating Water-Jet Nozzle

The nozzle investigated in this study consisted of four sections connected in sequence: an inlet cylindrical section, a converging section, a throat section, and an outlet diverging section. The total nozzle length, *l*, was 25.75 mm. The inlet cylindrical section had a length *l*_1_ of 3 mm and a diameter *d*_1_ of 7 mm and was designed to stabilize the incoming flow. The converging section had a length *l*_2_ of 11.75 mm and accelerated the flow. The downstream throat section had a length *l*_3_ of 3 mm. For the flat-slot nozzles, the throat cross-section was racetrack-shaped, consisting of a central rectangular region capped by two semicircles, to produce a uniform and concentrated jet. The divergence angle *θ* of the outlet diverging section was 60°. As the high-speed jet entered this section, strong wall shear, flow separation, and localized low-pressure regions developed near the walls, thereby promoting cavitation inception and cavitation-cloud development. A schematic of the flat-slot cavitating nozzle is shown in Figure 1.

To consistently describe the nozzle geometry and flow-field directions, the coordinate directions were defined with reference to the center of the throat cross-section. The height and width directions of the cross-section were defined as the *y*- and *x*-axes, respectively, whereas the jet-flow direction was defined as the *z*-axis, with its positive direction aligned with the jet. The coordinate origin *O* was located at the center of the nozzle inlet. The *y* = 0 plane was the central longitudinal *x*-*z* plane and was used to characterize flow-field distributions along the cross-sectional width direction. The *x* = 0 plane was the central longitudinal *y*-*z* plane and was used to characterize distributions along the cross-sectional height direction.

As shown in Figure 1b, the aspect ratio (AR) of the throat flow passage was defined as the ratio of its width *b* to its height *h*, i.e., AR = *b*/*h*. One circular reference nozzle (AR = 1) and four racetrack-shaped flat-slot nozzles with AR = 3, 5, 7, and 9 were considered. To isolate the effect of AR, the throat cross-sectional area of all five nozzles was maintained at 1.539 mm^2^, identical to that of the circular nozzle with a diameter of 1.4 mm. For the flat-slot nozzles, the throat width *b* and height *h* were determined according to the prescribed AR. For the racetrack-shaped cross-section consisting of a central rectangular region capped by two semicircles, the area *A* is given by *A* = *h*(*b* − *h*) + π*h*^2^/4. Combined with the definition of AR, this relationship was used to determine the corresponding width *b* and height *h* for each AR, as listed in Table 1. At a constant throat cross-sectional area, increasing AR produced a progressively flatter flow passage, accompanied by a reduction in hydraulic diameter and an increase in wetted perimeter. These geometric changes substantially affected the wall-shear characteristics and jet coherence.

At AR = 9, the nozzle throat had a distinctly elongated and narrow cross-section, with a minimum cross-sectional height of only 0.419 mm. Conventional machining methods therefore make it difficult to integrally fabricate the narrow flow passage and the complex three-dimensional transition from the circular inlet to the racetrack-shaped throat. Accordingly, selective laser melting (SLM) additive manufacturing was used to fabricate the five experimental nozzles. SLM enables the circular inlet passage, converging transition section, and racetrack-shaped throats with different ARs to be fabricated as an integral structure in a single forming process, thereby avoiding coaxiality and connection errors introduced by segmented machining. Previous studies have shown that AlSi10Mg fabricated using SLM exhibits a certain degree of resistance to cavitation erosion [31]. Considering that the nozzles in the present study were used for short-duration high-speed imaging experiments at a jet pressure of 10 MPa, AlSi10Mg was selected as the nozzle material. Photographs of the five fabricated nozzles are shown in Figure 2.

Considering that small-scale internal flow passages fabricated by selective laser melting (SLM) may exhibit dimensional deviations caused by powder melting, forming shrinkage, and post-processing, the actual throat dimensions of the five experimental nozzles were further measured to evaluate the agreement between the theoretical design dimensions and the fabricated dimensions. High-resolution images of the nozzle throat openings were acquired using a DSX-1000 digital microscope (Olympus Corporation, Tokyo, Japan). After spatial calibration, image-based measurements were used to determine the actual throat width bm and height hm, from which the actual aspect ratio ARm = bm/hm was calculated. Each dimension was measured three times, and the mean value was taken as the final result. The measured nozzle dimensions are listed in Table 2. As shown in Table 2, the measured aspect ratios of the five nozzles were close to their corresponding theoretical design values and maintained the same increasing trend as AR increased from 1 to 9. The actual aspect ratios of the AR = 1, 3, 5, 7, and 9 nozzles were 1.013, 3.036, 4.988, 7.055, and 8.933, respectively, corresponding to deviations from the theoretical values of approximately 1.31%, 1.21%, 0.25%, 0.78%, and 0.74%, respectively, with a maximum deviation of 1.31%. These results indicate good agreement between the principal throat geometric parameters of the five experimental nozzles and their design values, thereby providing an actual geometric basis for the experimental comparison of the cavitation characteristics of nozzles with different ARs.

### 2.2. Mesh Generation and Independence Verification

A mesh-independence analysis was conducted to reduce discretization errors and evaluate the sensitivity of the numerical results to mesh resolution [32]. For numerical simulations of cavitating water jets with geometrically symmetric computational domains, a half-domain model with a symmetry boundary condition can be used to reduce computational cost [33]. This study employed a three-dimensional computational model. Because the internal flow passage and external flow domain were symmetric about the *x* = 0 plane, only half of the computational domain on one side of the *x* = 0 plane was constructed to reduce computational cost. A symmetry boundary condition was applied on the *x* = 0 plane. The computational domain for the AR = 9 nozzle is shown in Figure 3. The external flow domain had a diameter of 20 mm and a length of 30 mm.

Five sets of unstructured tetrahedral meshes were generated for the half-domain model of the AR = 9 nozzle using ANSYS Workbench Meshing 2024 R2. Local mesh refinement was applied in the converging, throat, and diverging sections, as well as near the nozzle exit, where large velocity and pressure gradients were expected. The corresponding full-domain cell counts were 439,214, 900,626, 1,478,448, 1,966,820, and 2,477,956. Figure 4 presents the variations in the maximum vapor volume fraction and maximum jet velocity with the full-domain cell count. When the cell count reached 1,478,448, further mesh refinement resulted in relative changes of less than 0.1% in both quantities. Considering both computational accuracy and cost, the mesh-generation strategy corresponding to 1,478,448 full-domain cells was adopted for the simulations of all nozzle configurations.

To further evaluate the near-wall mesh resolution, the wall *y*+ values were determined for the five nozzle configurations, as summarized in Table 3. The mean *y*+ values for the final meshes of the AR = 1, 3, 5, 7, and 9 nozzles were 1.14, 1.46, 1.28, 2.02, and 2.30, respectively. The mean *y*+ values of all five nozzles were below 5 and remained at relatively low levels, indicating that the near-wall meshes used in this study provided high wall resolution. Picanço et al. [34] reported in a cavitation simulation using the SST *k*-*ω* model that *y* + <5 facilitates a relatively accurate resolution of the near-wall low-Reynolds-number region. Therefore, based on the mean wall *y*+ values, the near-wall mesh resolution of all five nozzles was generally sufficient to satisfy the near-wall resolution requirements of the SST *k*-*ω* model.

### 2.3. Governing Equations and Numerical Models

#### 2.3.1. Mixture Multiphase Model

Numerical simulations were performed to characterize the cavitating flow inside the nozzles. The Mixture model treats the liquid and vapor phases as an interpenetrating continuum with equivalent macroscopic properties. It solves the mixture momentum equation and the volume-fraction transport equation for the dispersed phase, while a slip-velocity model is introduced to account for relative motion between the phases [35]. Previous studies have reported satisfactory convergence and numerical stability of this model in multiphase-flow simulations [36]. Therefore, the Mixture model was adopted to simulate the cavitation process inside the nozzles. The governing equations are given below [37].

The mixture continuity equation is expressed as:

(1) $$\frac{\partial \rho_{m}}{\partial t}+\nabla \cdot \left(\rho_{m}v_{m}\right)=0,$$

(2) $$v_{m}=\frac{\sum_{n=1}^{2}\alpha_{n}\rho_{n}v_{n}}{\rho_{m}},$$

(3) $$\rho_{m}=\sum_{n-1}^{2}\alpha_{n}\rho_{n},$$

where *ρ*_m_ is the mixture density, *v*_m_ is the mass-averaged mixture velocity, *α_n_* is the volume fraction of phase *n*, *ρ_n_* is the density of phase *n*, *t* is time, and *v_n_* is the velocity of phase *n*.

The mixture momentum equation is expressed as:

(4) $$\frac{\partial }{\partial t}\left(\rho_{m}v_{m}\right)+\nabla \cdot \left(\rho_{m}v_{m}v_{m}\right)=-\nabla p+\nabla \cdot \left[\mu_{m}\left(\nabla v_{m}+{\left(\nabla v_{m}\right)}^{T}\right)\right]+\rho_{m}g+F+\nabla \cdot \left(\sum_{n=1}^{2}\alpha_{n}\rho_{n}v_{\mathrm{dr},n}v_{\mathrm{dr},n}\right),$$

(5) $$\mu_{m}=\sum_{n=1}^{2}\alpha_{n}\mu_{n},$$

(6) $$v_{\mathrm{dr},n}=v_{n}-v_{m},$$

where *p* is the pressure shared by all phases, *μ*_m_ is the dynamic viscosity of the mixture, g is the gravitational acceleration, *F* is the external body force, *v*_dr,*n*_ is the drift velocity of phase *n*, and *μ_n_* is the dynamic viscosity of phase *n*.

#### 2.3.2. SST *k*-*ω* Turbulence Model

The present study considers high-speed cavitating jets under submerged conditions. Pronounced near-wall velocity gradients, adverse pressure gradients, and localized flow separation occur in the converging, throat, and diverging sections of the nozzle. Meanwhile, the free-jet region downstream of the nozzle exit is characterized by strong shear-layer entrainment and vortex-structure evolution. The shear stress transport *k*-*ω* turbulence model (SST *k*-*ω*) integrates the near-wall boundary-layer treatment of the *k*-*ω* model with robust numerical performance for free-shear flows in the far field. Its shear-stress-transport correction also suppresses the nonphysical growth of turbulent viscosity, thereby enhancing predictive performance in flows involving adverse pressure gradients and separation. Accordingly, the SST *k*-*ω* formulation was employed in this study. The governing transport equations for *k* and *ω* can be written as follows [38,39]:

(7) $$\frac{\partial (\rho_{m}k)}{\partial t}+\frac{\partial }{\partial x_{i}}(\rho_{m}kv_{m,i})=\frac{\partial }{\partial x_{j}}\left(\Gamma_{k}\frac{\partial k}{\partial x_{j}}\right)+G_{k}-Y_{k}+S_{k}+G_{b},$$

(8) $$\frac{\partial (\rho_{m}\omega )}{\partial t}+\frac{\partial }{\partial x_{i}}(\rho_{m}\omega v_{m,i})=\frac{\partial }{\partial x_{j}}\left(\Gamma_{\omega }\frac{\partial \omega }{\partial x_{j}}\right)+G_{\omega }-Y_{\omega }+D_{\omega }+S_{\omega }+G_{\omega b},$$

(9) $$\Gamma_{k}=\mu_{m}+\frac{\mu_{t}}{\sigma_{k}},$$

(10) $$\Gamma_{\omega }=\mu_{m}+\frac{\mu_{t}}{\sigma_{\omega }},$$

where *v_m_*_,*i*_ is the component of the mass-averaged mixture velocity *v_m_* in the *i*-th coordinate direction; *G_k_* and *G_ω_* are the production terms for turbulent kinetic energy and specific dissipation rate, respectively; *Y_k_* and *Y_ω_* represent the dissipation of *k* and *ω* due to turbulence, respectively; Γ*_k_* and Γ*_ω_* are the corresponding effective diffusivities; *D_ω_* is the cross-diffusion term in the ω equation; *S_k_* and *S_ω_* are user-defined source terms in the *k* and *ω* equations, respectively; *G_b_* and *G_ωb_* represent the contributions of buoyancy to the transport equations for *k* and *ω*, respectively; and *σ_k_* and *σ_ω_* are the turbulent Prandtl numbers for *k* and *ω*, respectively.

The turbulent dynamic viscosity *μ_t_* is calculated as:

(11) $$\mu_{t}=\frac{\rho_{m}k}{\omega }\frac{1}{\max\left[\frac{1}{\alpha^{*}},\frac{SF_{2}}{a_{1}\omega }\right]}$$

(12) $$F_{2}=\tanh\left(\Phi_{2}^{2}\right)$$

(13) $$\Phi_{2}=\max\left[\frac{2\sqrt{k}}{0.09\omega y},\frac{500\mu_{m}}{\rho_{m}y^{2}\omega }\right]$$

where *α*^∗^ is the low-Reynolds-number correction coefficient for turbulent viscosity, *S* is the magnitude of the mean strain-rate tensor, *F*_2_ is the second blending function of the SST *k*-*ω* model, *a*_1_ is an empirical constant of the model, *Φ*_2_ is the dimensionless auxiliary variable used to evaluate *F*_2_, and *y* is the distance from the computational point to the nearest wall.

#### 2.3.3. Zwart–Gerber–Belamri Cavitation Model

In the submerged high-speed jet considered in this study, cavitation can develop once the local pressure drops below the saturation vapor pressure. Rapid liquid–vapor phase change involving vaporization and condensation then occurs, giving rise to cavitation clouds in the shear layer adjacent to the nozzle exit and in regions containing low-pressure vortical structures. Bubble growth and collapse are modeled using the Zwart–Gerber–Belamri (ZGB) formulation, which is based on a simplified Rayleigh–Plesset equation. Liquid–vapor phase change is incorporated into the model through mass-transfer source terms in the transport equation for vapor volume fraction. Owing to its relatively simple formulation and favorable numerical stability, the ZGB model was adopted to represent liquid–vapor mass transfer during jet cavitation.

The transport equation governing the vapor volume fraction takes the following form [40]:

(14) $$\frac{\partial }{\partial t}\left(\alpha_{v}\rho_{v}\right)+\nabla \cdot \left(\alpha_{v}\rho_{v}v_{v}\right)=R_{e}-R_{c},$$

where *α_v_* is the vapor volume fraction, *ρ_v_* is the vapor density, *v_v_* is the vapor-phase velocity, and *R*_e_ and *R*_c_ are the evaporation and condensation mass-transfer rates, respectively. These rates are defined as follows.

For *P* < *P_v_*:

(15) $$R_{e}=F_{\mathrm{vap}}\frac{3\alpha_{\mathrm{nuc}}\left(1-\alpha_{v}\right)\rho_{v}}{R_{B}}\sqrt{\frac{2}{3}\frac{P_{v}-P}{\rho_{l}}},$$

For *P* ≥ *P_v_*:

(16) $$R_{c}=F_{\mathrm{cond}}\frac{3\alpha_{v}\rho_{v}}{R_{B}}\sqrt{\frac{2}{3}\frac{P-P_{v}}{\rho_{l}}},$$

where *F*_vap_ and *F*_cond_ are the empirical calibration coefficients for evaporation and condensation, respectively; *α*_nuc_ is the nucleation-site volume fraction; *ρ*_l_ is the liquid density; *P* is the local static pressure; *P_v_* is the saturation vapor pressure; and *R_B_* is the bubble radius.

### 2.4. Boundary Conditions and Solution Settings

Numerical simulations of cavitating flow through the flat-slot nozzles at a submergence depth of 0.5 m were performed using ANSYS Fluent 2024 R2 (ANSYS, Inc., Canonsburg, PA, USA). The following boundary settings were prescribed for the computational domain. Fluent’s Operating Pressure was specified as 101,325 Pa. The inlet was defined as a pressure inlet with a pressure of 10 MPa. The outlet was prescribed as a pressure outlet at 5 kPa, corresponding to the hydrostatic pressure at a water depth of 0.5 m. Liquid water and water vapor were used as the working fluids. For water, the saturation vapor pressure was specified as 3540 Pa on an absolute-pressure basis. Liquid water had a density of 998.2 kg·m^−3^ and a dynamic viscosity of 0.001003 kg·m^−1^·s^−1^; the corresponding values for water vapor were 0.5542 kg·m^−3^ and 1.34 × 10^−5^ kg·m^−1^·s^−1^. A turbulence intensity of 5% was prescribed for both the inlet and the backflow. For the Zwart–Gerber–Belamri model, the parameter values were 0.001 mm for the bubble radius, 5 × 10^−4^ for the nucleation-site volume fraction, 50 for the evaporation coefficient, and 1 × 10^−2^ for the condensation coefficient.

A pressure-based transient solver was employed, and pressure and velocity were coupled by the pressure-implicit with splitting of operators (PISO) algorithm. The PRESTO! scheme was selected for pressure interpolation, QUICK was used for the volume-fraction equation, and second-order upwind discretization was adopted for the remaining equations. Gradients were evaluated using the cell-based least-squares method. To obtain an appropriate initial flow field for the transient calculations, a steady-state simulation was first performed until the monitored quantities, including the inlet and outlet mass flow rates, nozzle-exit velocity, and maximum vapor volume fraction across the computational domain, became approximately stable. The resulting steady-state field served as the initial state for the subsequent transient calculation.

To avoid the influence of time-step size on the computational results, a time-step-independence study was conducted using the AR = 9 nozzle as the representative case. Time-step sizes of 5 × 10^−6^, 2 × 10^−6^, 1 × 10^−6^, 5 × 10^−7^, and 2 × 10^−7^ s were examined using the same mesh, boundary conditions, and spatial discretization schemes. After the flow reached a statistically stationary state, the global maximum of the time-averaged vapor-volume-fraction field was evaluated over time intervals of equal duration. To quantitatively evaluate the sensitivity of the computational results to the time-step size, the result obtained using the smallest time-step size of 2 × 10^−7^ s was used as the reference value, and the relative differences between the results obtained using the other time-step sizes and the reference value were calculated, as summarized in Table 4.

As shown in Table 4, when the time-step size was reduced from 5 × 10^−6^ s to 2 × 10^−6^ s and below, the results remained within a narrow range, and their relative differences from the reference result obtained using 2 × 10^−7^ s were all below 0.2%. To balance numerical accuracy against computational cost, 1 × 10^−6^ s was adopted as the time-step size for the subsequent transient simulations. Within each time step, the convergence criterion required the scaled residuals for the continuity, momentum, turbulence, and vapor-volume-fraction equations to fall below 10^−4^. The maximum number of inner iterations per time step was set to 30. The total physical simulation time for each case was 25 ms, corresponding to 25,000 time steps. To exclude the initial flow-development stage, the results obtained during the first 5 ms were discarded, and the velocity, vapor volume fraction, and other flow quantities were time-averaged over t = 5–25 ms.

### 2.5. High-Speed Imaging Method

The cavitating-jet visualization facility comprised two main subsystems: a high-pressure water-jet system and a high-speed imaging system, as illustrated in Figure 5. The high-pressure water-jet subsystem mainly included a cavitation test tank, nozzle, nozzle holder, overflow bucket, water-supply tank, high-pressure pump assembly, and control unit. The imaging subsystem comprised a high-speed camera, a computer, and an illumination device. A CHC16/50 S-L triplex plunger pump (UDOR S.p.A., Rubiera, Italy) rated at 50 MPa with a maximum flow rate of 16 L/min was used to provide a stable output at the target pressure of 10 MPa. The pump outlet was connected to the nozzle holder via a high-pressure hose, while the test nozzle was mounted in the holder and immersed in the cavitation test tank. An overflow port was provided in the tank sidewall. Continuous overflow was maintained during each test to keep the water level constant, thereby ensuring a stable submergence depth of 0.5 m at the nozzle exit.

A Y4-S1 high-speed camera (Integrated Design Tools, Inc., Pasadena, CA, USA), equipped with an AF Nikkor 50 mm f/1.8D fixed-focal-length lens (Nikon Corporation, Tokyo, Japan), was used. The maximum frame rate of the camera was 130,000 fps. Illumination was provided by a 300 W light-emitting diode panel with a color temperature of 6500 K. The light was diffused through a light-diffusing plate and projected laterally across the jet to produce a relatively uniform illuminated region. To enhance the contrast between the cavitation cloud and the background, a black backdrop was placed behind the jet, opposite the camera. The numerous liquid–vapor interfaces within the cavitation cloud scattered, refracted, and locally reflected the incident light, causing the cloud to appear as a bright region against the black background and thereby producing images of white cavitation clouds [41].

Experiments were conducted sequentially using the five nozzles with AR = 1, 3, 5, 7, and 9. During the experiments, the *y*-axis of the nozzle was aligned parallel to the optical axis of the camera, such that the recorded images represented the projection of the cavitation cloud along the *y*-direction onto the *x*-*z* plane. Before each experiment, the cavitation test tank was filled until overflow occurred. After a constant water level had been confirmed, the nozzle inlet pressure was adjusted to 10 MPa. The system was operated steadily for approximately 30 s before high-speed recording was initiated. The imaging parameters were set to a recording rate of 10,000 fps, a lens aperture of f/1.8, and an image resolution of 1280 × 800 pixels. For each nozzle configuration, images were continuously acquired for 0.2 s, corresponding to 2000 frames, to capture multiple cycles of transient cavitation-cloud evolution.

To assess experimental repeatability, two independent tests were conducted for each nozzle under identical conditions. The pump assembly was restarted, and the camera was refocused and spatially calibrated before each test. In the time-averaged gray-level contour maps obtained from the two repeated tests, the relative deviations in the maximum length and width of the high-gray-level region, defined over a normalized gray-level range of 0.9–1.0, were both below 5%. These results indicated satisfactory stability and repeatability of the experimental measurements.

To reduce the influence of transient cavitation-cloud shedding and local illumination fluctuations on morphological characterization, the 2000 images acquired for each nozzle configuration were first converted to grayscale and time-averaged to obtain a time-averaged gray-level image [42]. To place the results for all nozzle configurations on a common scale, the dimensionless gray-level analysis method previously applied to cavitating jets was adopted [43]. The time-averaged gray-level data from all five configurations were treated as a single dataset, and a common global minimum and maximum were used for linear normalization. The normalized gray level *I_i_*_,*j*_ was calculated as follows:

(17) $$I_{i,j}=\frac{G_{i,j}-G_{\min}}{G_{\max}-G_{\min}}$$

where *G_i_*_,*j*_ is the gray level of the pixel in row *i* and column *j*, and *G*_min_ and *G*_max_ are the global minimum and maximum gray levels, respectively, across all operating conditions. Because the same normalization limits were applied to all cases, the normalized gray levels for different ARs were expressed on a common scale and could therefore be used to compare relative differences in the optical responses of the cavitation clouds.

## 3. Results

### 3.1. Effect of Aspect Ratio on Jet Velocity Distribution

Figure 6 shows the numerically obtained variation in the time-averaged jet centerline velocity with axial distance for different aspect ratios. Overall, the centerline velocity exhibited similar axial evolution for all nozzle configurations, including an initial low-velocity region, rapid acceleration through the converging section, maintenance of a high velocity near the nozzle exit, and rapid decay in the external flow field. However, noticeable differences occurred within the throat and diverging sections, corresponding to the axial range of 14.75–25.75 mm. In the inlet cylindrical section (0–3 mm), the flow-passage area remained constant, and the centerline velocity varied only slightly. After the flow entered the converging section (3–14.75 mm), the cross-sectional area gradually decreased, causing the fluid to accelerate and the centerline velocity to rise rapidly to its peak value. Although the throat cross-sectional area was identical for all configurations, increasing AR altered the cross-sectional width and height, thereby increasing the wetted perimeter and reducing the hydraulic diameter. These changes affected wall friction, local velocity gradients, and the development of the near-exit shear layer. Consequently, slight differences were observed in the peak centerline velocities among the nozzle configurations. For AR = 1, 3, 5, 7, and 9, the peak time-averaged centerline velocities were 142.35, 142.30, 142.62, 143.02, and 143.02 m/s, respectively. The relative differences among the configurations were all less than 1%, indicating that AR had only a limited effect on the peak centerline velocity.

After the flow entered the throat section (14.75–17.75 mm), the centerline velocity profiles began to diverge. For AR = 1 and AR = 3, the jet velocity remained nearly constant within the throat, indicating that the flow maintained centerline momentum relatively well in this region. For AR ≥ 5, the centerline velocity exhibited a decreasing trend within the throat, with a more pronounced decline at higher ARs. Upon entering the diverging section (17.75–25.75 mm), the centerline velocity decreased to varying degrees because of flow expansion, boundary-layer development, and viscous dissipation. As AR increased, the throat height *h* decreased, and the hydraulic diameter was reduced, resulting in greater wall friction and steeper velocity gradients. Consequently, momentum dissipation within the throat and diverging sections increased, leading to a more pronounced reduction in centerline velocity. Nevertheless, the high-speed jet core retained sufficient momentum to maintain a relatively high velocity in the downstream portion of the diverging section and near the nozzle exit. After the jet entered the external flow field at axial positions beyond 25.75 mm, confinement by the nozzle walls disappeared, and momentum exchange and turbulent mixing between the jet and the surrounding quiescent water intensified. The centerline velocity therefore continued to decrease with increasing axial distance. These results indicate that AR primarily affected momentum transport and dissipation within the throat and diverging sections by altering the characteristic dimensions of the nozzle cross-section, thereby modifying the velocity distribution near the nozzle exit and in the external flow field.

Figure 7 presents the time-averaged velocity distributions in the *y* = 0 plane obtained from the numerical simulations for the different nozzle configurations. Under identical inlet-pressure and throat-area conditions, a distinct high-speed jet core formed near the end of the converging section, throughout the throat, and near the entrance of the diverging section for all nozzles, indicating that the fluid was sufficiently accelerated inside the nozzle. For the AR = 1 and AR = 3 nozzles, the high-speed region exhibited an elongated distribution and extended relatively far downstream of the nozzle exit, indicating stronger axial momentum retention at lower ARs. As AR increased, the spatial structure of the velocity field gradually changed. For AR ≥ 5, the high-speed core exhibited a distinct wedge-shaped distribution near the nozzle exit. Compared with the lower-AR nozzles, the higher-AR nozzles produced a more pronounced expansion of the high-speed region along the cross-sectional width direction, i.e., the *x*-direction. At the same time, the axial extent of the high-speed core in the external flow field decreased, and the onset of velocity decay shifted upstream. Thus, although higher-AR nozzles produced more pronounced lateral spreading near the exit, they also intensified momentum exchange between the jet and the surrounding fluid, thereby reducing downstream axial-momentum retention.

Taken together, Figure 6 and Figure 7 show that AR had only a limited effect on the peak centerline velocity but markedly affected the spatial structure of the velocity field, the axial extent of the high-speed core, and the distribution of the near-exit shear layer. At lower ARs, the jet velocity field was more strongly concentrated in the axial direction, and the high-speed core extended farther downstream. At higher ARs, the jet exhibited greater near-exit lateral spreading and more rapid downstream velocity decay. These results demonstrate that, at a constant throat cross-sectional area, the effect of AR was reflected not only in the peak velocity but also in the redistribution of jet momentum between the axial and *x*-directions.

### 3.2. Effect of Aspect Ratio on Cavitation Performance

Based on the numerical simulation results, the vapor volume fraction at each spatial location in the complete fluid domain was time-averaged over the statistical interval of 5–25 ms to obtain the time-averaged vapor-volume-fraction field. The spatial global maximum was then extracted from this time-averaged field and defined as the global maximum of the time-averaged vapor-volume-fraction field. Figure 8 shows the variation in the global maximum of the time-averaged vapor-volume-fraction field for different aspect ratios. As AR increased, this quantity exhibited a nonmonotonic trend, initially decreasing and then recovering. For AR = 1, 3, 5, 7, and 9, the corresponding values were 94.86%, 93.17%, 93.74%, 93.89%, and 94.23%, respectively. The minimum value occurred at AR = 3, representing a decrease of approximately 1.69 percentage points relative to that at AR = 1. As AR further increased to 5, 7, and 9, the global maximum gradually recovered. At AR = 9, it reached the highest value among the flat-slot nozzles (AR ≥ 3), although it remained slightly below that of the circular nozzle at AR = 1. Notably, the overall variation in this peak quantity was relatively small, indicating limited sensitivity to AR. Therefore, the global maximum alone cannot fully characterize the effect of nozzle AR on cavitation development. The spatial and axial distributions of the vapor volume fraction were consequently examined to provide a more comprehensive assessment of the cavitation characteristics.

Figure 9 presents the time-averaged vapor-volume-fraction distributions in the *y* = 0 plane obtained from the numerical simulations for the different nozzle configurations. At AR = 1, regions of high vapor volume fraction were mainly located on both sides of the nozzle diverging section, forming two distinct localized zones. This distribution indicates that cavitation occurred primarily within the near-wall separation regions close to the nozzle exit and within the shear layers along the jet periphery. At AR = 3, the high-vapor-volume-fraction regions remained concentrated near the walls of the diverging section; however, their extent along the two sidewalls in the *x*-direction decreased relative to that at AR = 1, and the local peak values were also reduced. Meanwhile, a low-vapor-volume-fraction region began to develop near the jet center.

As AR increased further to AR ≥ 5, the high-vapor-volume-fraction regions near the two sidewalls in the *x*-direction continued to contract, whereas the cavitation cloud in the jet center became markedly more pronounced and formed a continuous axially developing cavitating region downstream of the nozzle exit. In particular, at AR = 7 and AR = 9, the high-vapor-volume-fraction region exhibited a triangular distribution that was wider upstream and gradually narrowed downstream. This pattern indicates that higher ARs altered the spatial mode of cavitation development in the *y* = 0 plane, causing the cavitating region to evolve from localized near-wall concentrations into a continuous distribution within the jet core.

Figure 10 shows the time-averaged vapor-volume-fraction distributions in the *x* = 0 plane obtained from the numerical simulations. As indicated by Figure 9 and Figure 10, the cavitation clouds generated by the flat-slot nozzles were primarily concentrated in the near-wall regions on both sides along the *y*-direction. With increasing AR, the axial extent of the cavitation cloud along the *z*-direction increased, whereas its width along the *y*-direction decreased. At AR = 1, the circular nozzle produced a relatively symmetric jet cross-section. The cavitation cloud was mainly concentrated around the jet periphery, while the vapor volume fraction in the jet center remained low.

At AR = 3, the increase in AR reduced the throat height *h*, thereby decreasing the separation between the cavitation clouds generated near the two opposing walls in the *y*-direction. These clouds began to contact and overlap near the jet center, producing a low-intensity cavitation cloud in the central region. At AR = 5, the throat height *h* decreased further, and the near-wall cavitation clouds on both sides became nearly connected, resulting in a further increase in the vapor volume fraction in the jet center. At AR = 7, the high-vapor-volume-fraction regions near the two opposing walls in the *y*-direction began to merge. This connection became more pronounced at AR = 9, leading to greater continuity of the cavitation cloud within the jet core.

As shown in Figure 8, Figure 9 and Figure 10, AR had only a limited effect on the global maximum of the time-averaged vapor-volume-fraction field but substantially altered the spatial distribution and morphology of the cavitating regions. As AR increased, the near-wall high-vapor-volume-fraction regions on both sides in the *x*-direction gradually weakened and contracted. Meanwhile, as the throat height *h* decreased, the cavitation clouds generated near the two opposing walls in the *y*-direction moved progressively toward the jet center, resulting in an increase in central cavitation intensity. The separation between these near-wall cavitation clouds was consequently reduced, causing them to converge toward the jet center and ultimately form a relatively continuous cavitating region within the jet core. These results indicate that, at a constant throat cross-sectional area, variations in AR substantially altered the wall confinement and spatial distribution of the cavitating regions in the two cross-sectional directions, thereby further affecting the mutual approach, overlap, and axial development of the cavitation clouds.

To further quantitatively characterize the extent to which cavitation developed toward the jet-center region under different AR conditions, the time-averaged vapor volume fraction along the geometric centerline of the nozzle (*x* = 0, *y* = 0) was extracted for comparison. Figure 11 shows the numerically obtained axial distributions of the time-averaged vapor volume fraction along the jet centerline for the flat-slot nozzles (AR ≥ 3). For all flat-slot nozzles, the centerline vapor volume fraction first increased and then decreased with axial distance. Both its peak value and axial extent increased with AR. At AR = 3, 5, 7, and 9, the peak time-averaged centerline vapor volume fractions were 39.26%, 55.66%, 67.65%, and 77.84%, respectively. The peak value at AR = 9 was 38.58 percentage points higher than that at AR = 3, corresponding to a relative increase of 98.27%. Together with the distributions shown in Figure 9 and Figure 10, these results indicate that, as AR increased, the separation between the near-wall cavitating regions on both sides progressively decreased, and these regions approached and overlapped more markedly toward the jet center at higher ARs. Consequently, both the time-averaged vapor volume fraction and the axial cavitation extent along the centerline increased with AR. This behavior further highlights the influence of nozzle AR on the spatial structure and development of the cavitation cloud.

Overall, variations in AR not only altered the spatial distribution of cavitating regions in the two cross-sectional directions but also produced pronounced directional differences in cavitation development near different walls of the diverging section. At a constant throat cross-sectional area, increasing AR increased the cross-sectional width *b* while decreasing the height *h*, resulting in opposite changes in wall confinement along the *x*- and *y*-directions. As shown in Figure 9, the near-wall high-vapor-volume-fraction regions on both sides in the *x*-direction gradually contracted with increasing AR. In contrast, Figure 10 shows that the near-wall cavitating regions on both sides in the *y*-direction exhibited more pronounced axial development and mutual approach as AR increased, progressively forming a more continuous cavitation distribution at higher ARs. During this evolution, AR = 3 can be regarded as a transitional condition from a circular cross-section to a distinctly flattened cross-section. When AR increased from 1 to 3, the original approximately axisymmetric cavitation-distribution pattern was disrupted, and the high-vapor-volume-fraction regions near the walls in the *x*-direction weakened markedly, whereas the enhanced near-wall cavitation characteristics in the *y*-direction observed at higher ARs had not yet fully developed. This redistribution of near-wall cavitation corresponded to the lowest global maximum of the time-averaged vapor-volume-fraction field at AR = 3 and may be an important reason for the observed nonmonotonic variation. As AR further increased to 5, 7, and 9, the near-wall cavitating regions in the *y*-direction progressively intensified and extended downstream, accompanied by an increase in the local high-vapor-volume-fraction level, corresponding to the gradual recovery of the global maximum of the time-averaged vapor-volume-fraction field shown in Figure 8.

### 3.3. High-Speed Imaging Results

During the high-speed imaging experiments, 2000 instantaneous cavitating-jet images were continuously acquired for each nozzle configuration. According to the image-processing method described in Section 2.5, the raw images directly acquired by the high-speed camera were converted to grayscale, time-averaged, and uniformly normalized to obtain the time-averaged grayscale images and their corresponding contour maps for the five nozzle configurations, as shown in Figure 12. To prevent low-gray-level regions from interfering with the identification of cavitation-cloud morphology, data within the normalized gray-level range of 0–0.1 were excluded from the contour maps in Figure 12b. The gray level itself is not equivalent to the local vapor volume fraction, and their relationship is affected by factors such as the bubble-size distribution, multiple scattering, and line-of-sight integration. However, under identical camera settings, illumination conditions, and image-processing procedures, a higher normalized time-averaged gray level generally corresponds to a stronger optical response resulting from the scattering, refraction, and local reflection of light by bubble populations. Therefore, this quantity can still be used to characterize the macroscopic morphology, spatial distribution, and optical-response characteristics of cavitation clouds in two-dimensional projections under different operating conditions.

To quantitatively characterize the macroscopic projected morphology of the cavitation clouds, regions with normalized time-averaged gray levels of 0.9–1.0 were defined as high-gray-level regions. Image-Pro Plus 6.0 was used to determine their projected area *S_g_*, maximum width *W_g_*, and maximum length *L_g_*, as summarized in Table 5. As AR increased from 1 to 9, all three parameters first decreased and then increased, reaching their minimum values at AR = 3. This nonmonotonic trend was similar to that of the global maximum of the time-averaged vapor-volume-fraction field obtained numerically. For the flat-slot nozzles, the three geometric parameters increased progressively as AR increased from 3 to 9. At AR = 9, the projected area, maximum width, and maximum length reached 25.14 mm^2^, 2.64 mm, and 11.64 mm, respectively. By comparison, the high-gray-level region generated by the circular nozzle at AR = 1 had a maximum length of 17.40 mm, indicating a greater axial extent of the high-gray-level cavitation-cloud projection.

The gray levels along the jet centerline were extracted from the time-averaged images for all five cases, and the resulting axial distributions are shown in Figure 13. At AR = 1, 3, 5, 7, and 9, the corresponding peak time-averaged centerline gray levels were 0.967, 0.918, 0.920, 0.958, and 0.992, respectively. The peak time-averaged centerline gray level of the AR = 9 nozzle was 0.992, which was higher than the value of 0.967 for the circular nozzle at AR = 1. For the flat-slot nozzles (AR ≥ 3), the peak centerline gray level generally increased with AR, indicating a stronger time-averaged optical response and a more pronounced cavitation-cloud projection in the jet-center region at higher ARs. This trend was consistent with the numerically predicted variation in the centerline vapor volume fraction, indicating consistency between the two characterization methods in terms of the AR-dependent trend.

Overall, the high-speed imaging results showed that the projected area, maximum length, and maximum width of the high-gray-level region exhibited a nonmonotonic variation with AR, first decreasing and then increasing. This trend was similar to that of the global maximum of the time-averaged vapor-volume-fraction field. For the flat-slot nozzles, the peak time-averaged centerline gray level generally increased with AR, consistent with the numerical trend in the time-averaged vapor volume fraction along the jet centerline. These results provided experimental support for the numerical predictions from two complementary perspectives: the macroscopic projected morphology of the cavitation cloud and the optical response along the jet centerline. Because the grayscale distribution obtained by high-speed imaging represents a two-dimensional optical projection of the cavitation cloud along the camera line of sight and does not have a direct quantitative correspondence with the local vapor volume fraction on a specific numerical plane, the high-speed imaging experiments in this study were used primarily to compare the macroscopic projected morphology, optical response in the central region, and their AR-dependent trends, and to provide qualitative validation of the numerical results.

## 4. Discussion and Conclusions

### 4.1. Discussion

This study combined numerical simulations with high-speed imaging experiments to investigate the effects of nozzle aspect ratio on the velocity field and cavitation-cloud distribution of submerged cavitating water jets. The results showed that, at a constant throat cross-sectional area, variations in AR had only a limited effect on the maximum centerline velocity but markedly altered the near-exit velocity-field structure, the spatial distribution of cavitation, and cavitation development in the jet-center region. In particular, for the flat-slot nozzles, the vapor volume fraction in the central region increased markedly with AR. This finding indicates that AR affects not only the cross-sectional geometry of the nozzle but also the redistribution of jet momentum and cavitating regions within the flow field.

The present results are generally consistent with previous studies of noncircular nozzles. Yin et al. [25] reported that an elliptical nozzle with AR = 4 suppressed internal cavitation relative to a circular nozzle while increasing the spanwise spreading range and improving atomization characteristics. In the present study, the numerical results showed that the global maxima of the time-averaged vapor-volume-fraction field at AR = 3 and AR = 5 were lower than the corresponding value for the circular nozzle; the high-gray-level projected regions obtained by high-speed imaging also exhibited a corresponding reduction for the lower-AR flat-slot nozzles. These results indicate that cross-sectional flattening can substantially alter the spatial distribution of cavitation, and the variation in local peak values observed numerically is generally consistent with the trend in internal cavitation reported by Yin et al. [25]. Wu et al. [28] numerically found that the vapor volume fraction in the jet-center region of a flat nozzle was greater than that of a conventional circular nozzle. Similarly, the higher-AR flat-slot nozzles examined here exhibited a greater vapor volume fraction in the jet center and more pronounced axial cavitation development than the circular nozzle. In addition, the simulation results for the circular reference nozzle (AR = 1) showed that the cavitation cloud was concentrated primarily near the jet periphery and adjacent to the walls, consistent with the observation by Gopalan et al. [44] that cavitation clouds in circular jets are generally concentrated around the outer region of the jet. These comparisons demonstrate that the cavitation-distribution patterns obtained in this study are consistent with previously reported trends and thereby provide additional support for the physical reasonableness of the numerical results.

From an engineering perspective, the cavitation-distribution characteristics identified in this study provide useful guidance for the flow-field design of nozzles intended for flake graphite exfoliation. Shen et al. [45] achieved the mechanical exfoliation of crystalline graphite through jet-cavitation experiments and suggested that the microjets and shock waves generated by cavitation-bubble collapse, together with the induced stress effects, constitute important mechanical mechanisms promoting graphite-layer separation. Therefore, the spatial distribution of cavitation may further affect the extent to which graphite particles are subjected to microjet impacts within the jet. The present results showed that higher-AR flat-slot nozzles caused the cavitation-cloud distribution to become progressively concentrated toward the jet center and markedly increased the vapor volume fraction in the central region. At AR = 9, the peak centerline vapor volume fraction reached 77.84%, representing a relative increase of 98.27% compared with that at AR = 3. This centrally concentrated cavitation distribution may allow microjet impacts to act more directly on graphite particles passing through the jet center. Meanwhile, the reduced throat height may enhance velocity gradients and frictional shear in the height direction. The combined action of these effects may facilitate preferential exfoliation of graphite particles along their basal planes. However, this interpretation requires verification through subsequent graphite exfoliation experiments, and the present study provides only a flow-field-level design basis.

### 4.2. Limitations and Future Work

This study has several limitations in terms of numerical simulation, experimental validation, and nozzle-manufacturing characterization. To reduce computational cost, a half-domain model with a symmetry boundary condition was adopted based on the geometric symmetry of the nozzle and computational domain. This treatment is suitable for analyzing the time-averaged velocity field, time-averaged vapor-volume-fraction distribution, and relative variations among different ARs considered in this study; however, the symmetry boundary condition may restrict potential asymmetric turbulent structures and cavitation-cloud shedding behavior in the transient flow. Therefore, the present study focused primarily on the time-averaged flow field and cavitation-distribution characteristics and did not further quantitatively analyze transient asymmetric cavitation-cloud evolution. The high-speed imaging experiments were used mainly to validate cavitation-cloud morphology and grayscale-distribution trends, whereas the actual erosion performance and flake graphite exfoliation performance were not directly measured. In addition, transient high-speed images were not further used to quantitatively extract the cavitation-cloud shedding periods and dominant frequencies under different AR conditions. Regarding nozzle manufacturing, the measured width and height of the throat opening were used to verify the agreement between the actual and theoretical ARs of the different nozzles. However, owing to the difficulty of measuring small-scale enclosed internal flow passages, the three-dimensional profile error and internal surface morphology of the transition region from the circular inlet to the racetrack-shaped throat were not directly quantified. Local dimensional deviations and internal-wall surface characteristics introduced by SLM may affect flow separation, local pressure distributions, and cavitation development.

Future work can be conducted in several directions. Full-domain simulations combined with higher-fidelity unsteady approaches, such as large-eddy simulation (LES), can be employed to elucidate cavitation-cloud shedding, collapse, and vortex-structure evolution. Internal geometric characterization methods, such as X-ray computed tomography, can be used to reconstruct the actual three-dimensional internal flow passages of the nozzles and further evaluate the effects of manufacturing deviations on the cavitating flow field. Edge detection and grayscale-signal spectral analysis can be applied to extract cavitation-cloud shedding periods and dominant frequencies under different AR conditions. By varying the inlet pressure and introducing target-surface erosion and flake graphite exfoliation experiments, future studies can investigate the effective working distance, suitable stand-off distance, and actual material-processing performance, as well as evaluate the long-term service stability of the nozzles under cavitation. In addition, for abrasive water-jet applications, a solid-particle phase can be further coupled with the present liquid–vapor two-phase flow-field model to investigate abrasive transport and liquid–solid interactions under cavitating conditions.

### 4.3. Conclusions

Numerical simulations and high-speed imaging experiments were used to examine how the nozzle aspect ratio affects the velocity field and cavitation-cloud distribution of submerged cavitating water jets at a constant throat cross-sectional area. The main findings are summarized below.

(1) The maximum centerline velocity varied by less than 1% among the five nozzles, indicating that AR had little influence on the peak velocity under the same inlet pressure and throat area. However, AR markedly altered the spatial structure of the velocity field near the nozzle exit. As AR increased, the near-exit high-speed core spread more in the lateral direction, the axial extent of the high-speed core in the external flow field decreased, and the centerline velocity decayed more rapidly.
(2) The global maximum of the time-averaged vapor volume fraction varied only slightly with AR, but the spatial distribution of cavitation differed considerably. As AR increased, the near-wall high-vapor-volume-fraction regions on both sides in the *x*-direction weakened and contracted in the *y* = 0 plane; meanwhile, in the *x* = 0 plane, the near-wall cavitation clouds on both sides in the *y*-direction progressively moved toward the jet center and overlapped, ultimately forming a more continuous cavitating region within the jet core.
(3) For the flat-slot nozzles, increasing AR enhanced cavitation development in the jet-center region. The peak time-averaged centerline vapor volume fraction increased monotonically from 39.26% at AR = 3 to 77.84% at AR = 9, corresponding to a relative increase of 98.27%. These results indicate that higher-AR flat-slot nozzles favor the development of a more continuous and concentrated cavitating region in the jet center.
(4) High-speed imaging showed that the projected area, maximum width, and maximum length of the high-gray-level region varied nonmonotonically with AR, following a trend similar to that of the global maximum vapor volume fraction. For the flat-slot nozzles, the peak time-averaged centerline gray level generally increased with AR, consistent with the numerically predicted centerline vapor volume fraction trend. The experiments therefore provide qualitative support for the physical reasonableness of the numerical predictions.

Overall, the present numerical and experimental results clarify the effects of nozzle AR on jet structure and cavitation characteristics and provide a flow-field basis for the structural design of flat-slot cavitating nozzles and the selection of candidate nozzle parameters for subsequent flake graphite exfoliation studies.

## Figures and Tables

**Figure 1:**
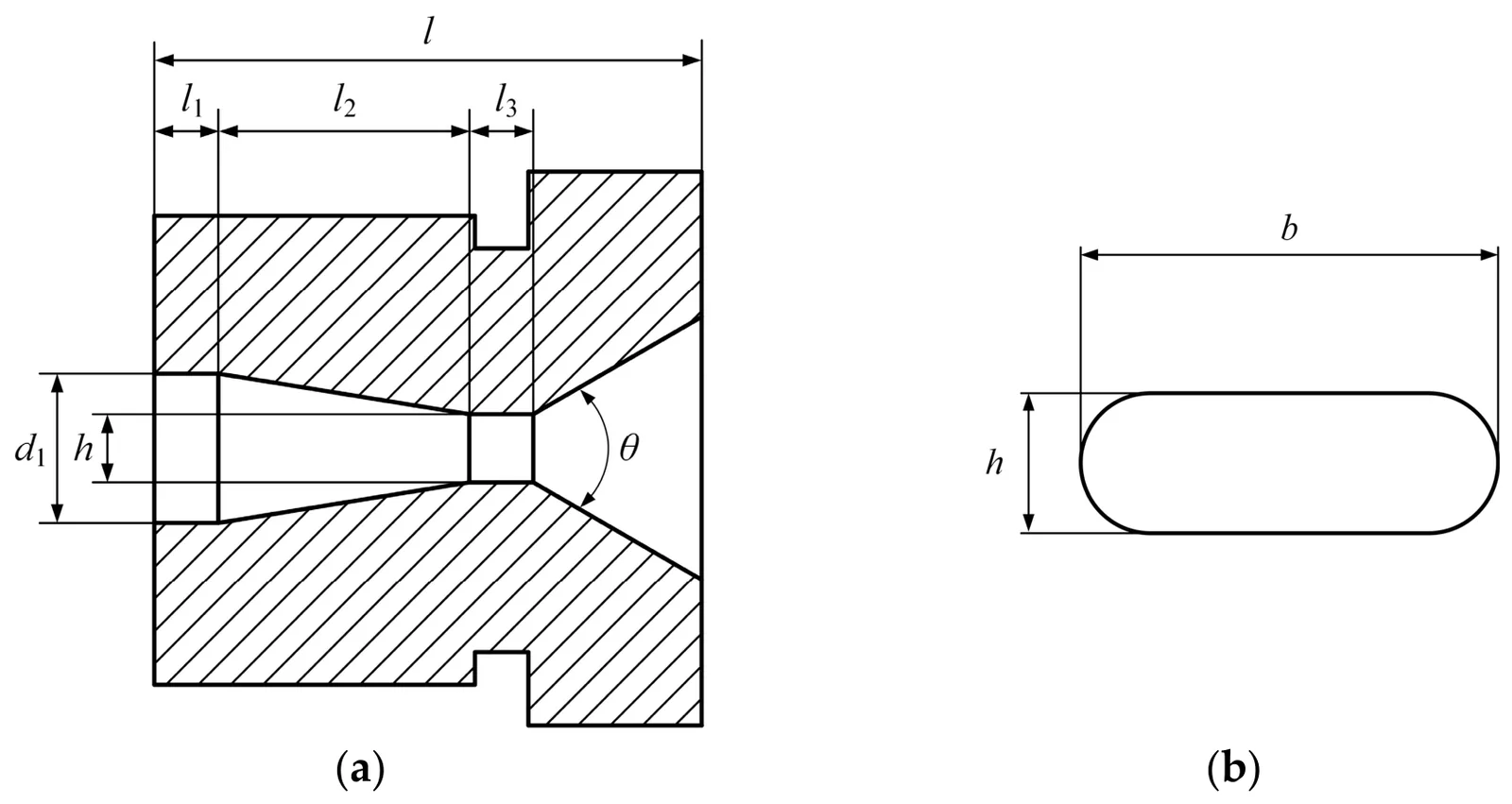
Schematic of the flat-slot cavitating nozzle geometry. (**a**) Sectional view in the central *x* = 0 plane; (**b**) Cross-sectional shape of the throat flow passage.

**Figure 2:**
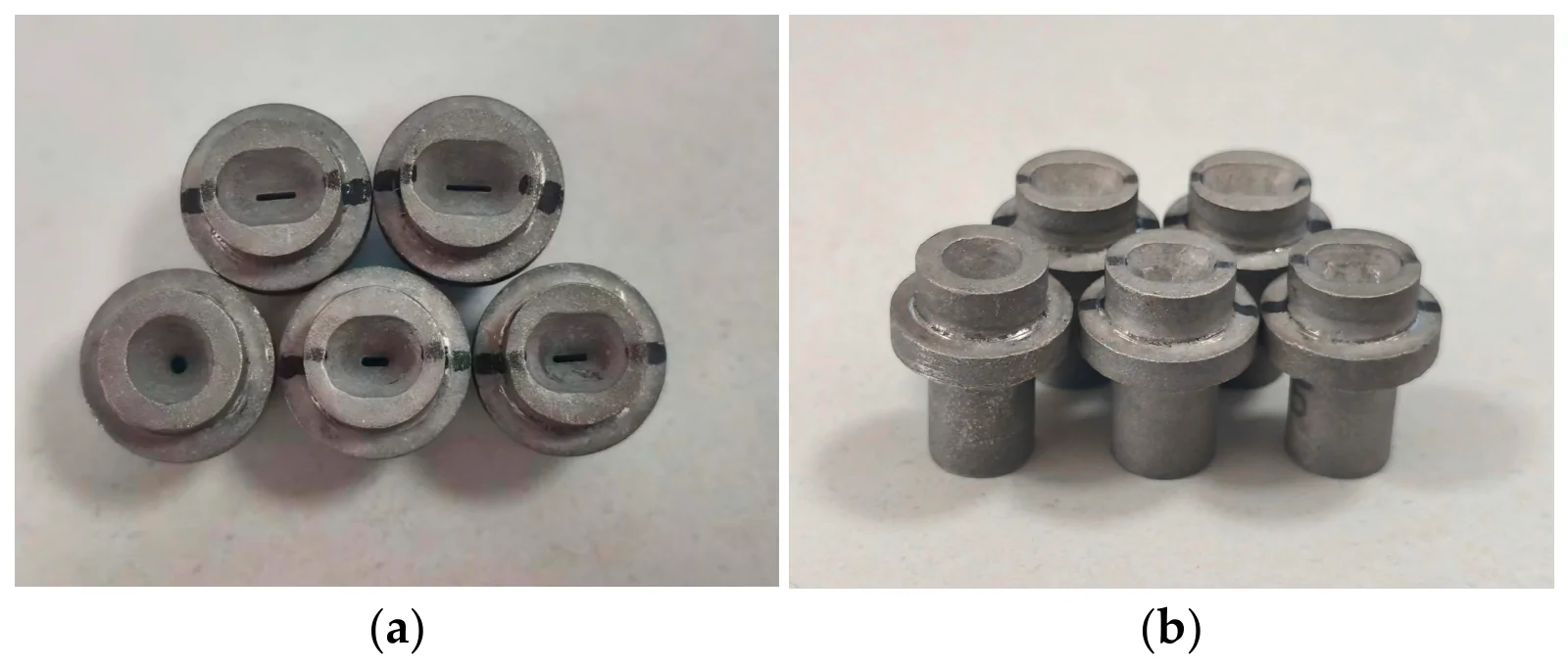
Photographs of the fabricated nozzles. (**a**) top view; (**b**) side view.

**Figure 3:**
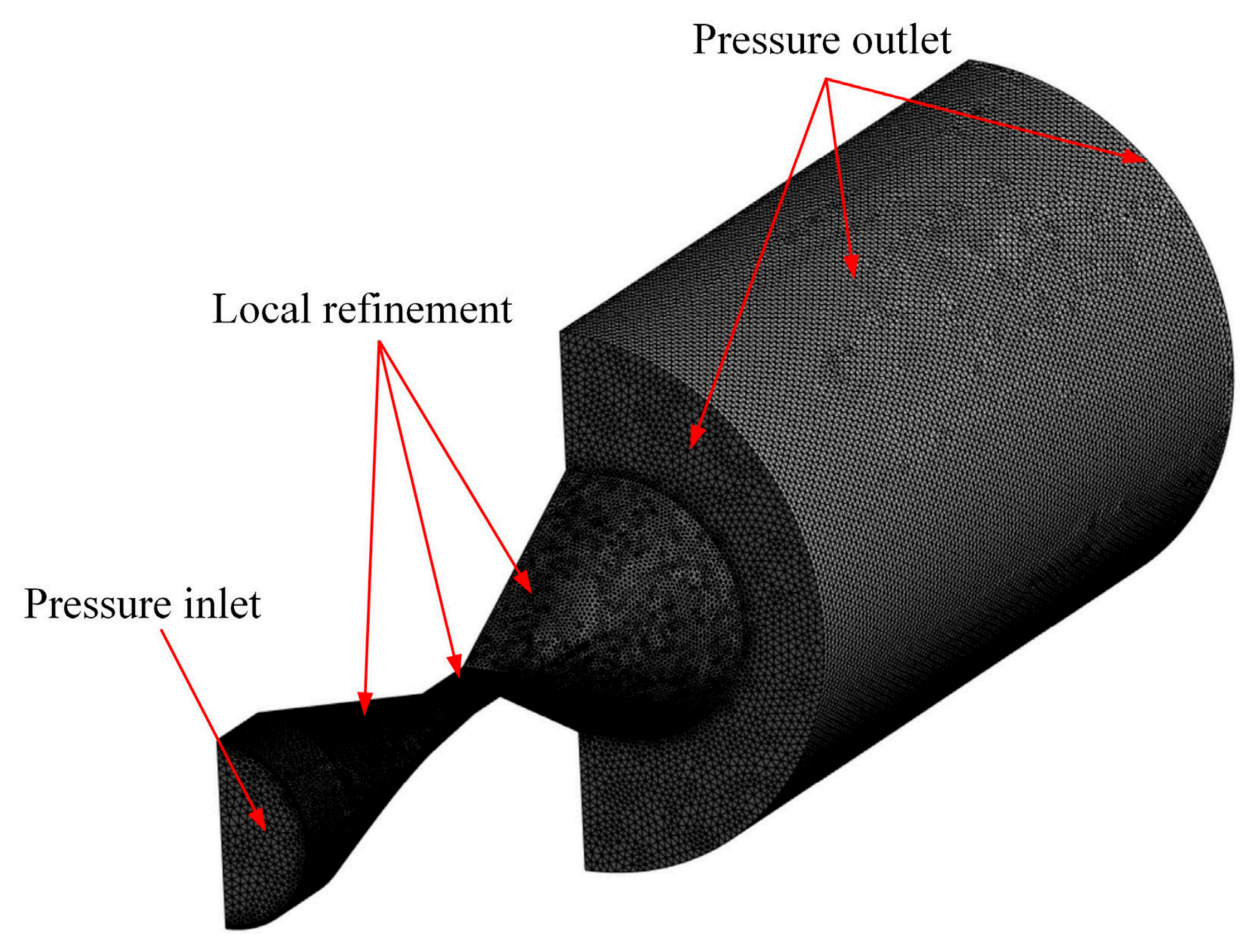
Geometric model of the computational flow domain.

**Figure 4:**
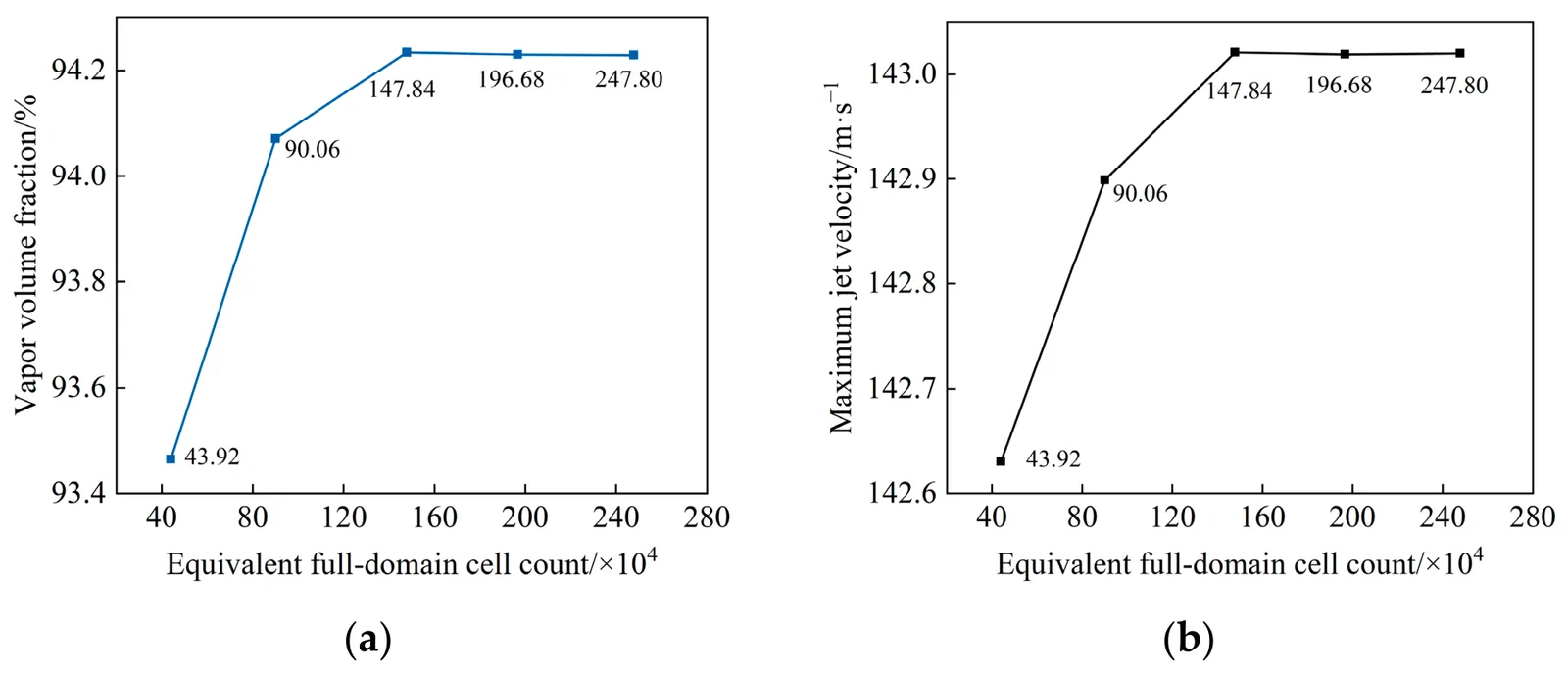
Mesh-independence analysis. (**a**) Maximum vapor volume fraction; (**b**) Maximum jet velocity.

**Figure 5:**
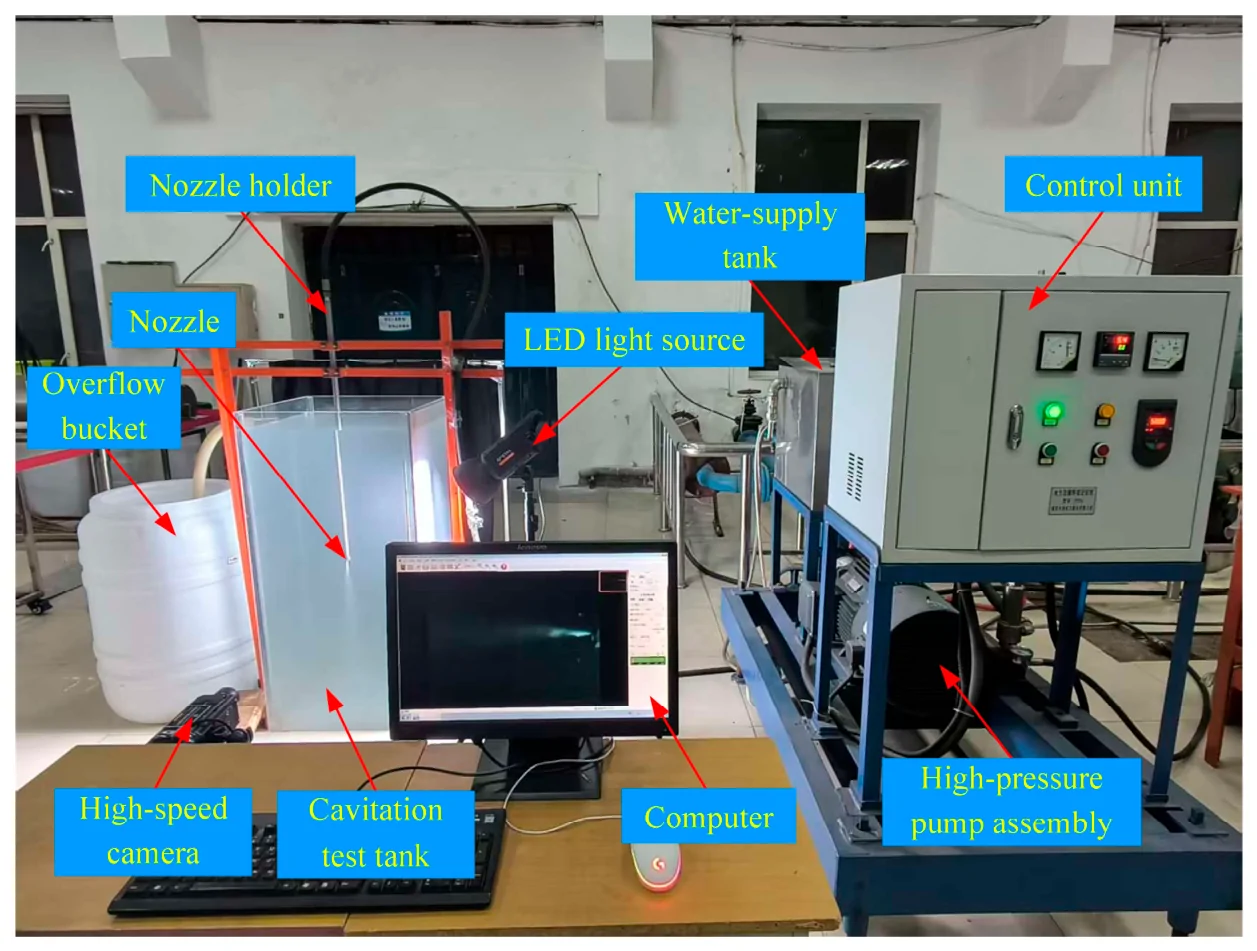
Schematic of the cavitating water-jet visualization facility.

**Figure 6:**
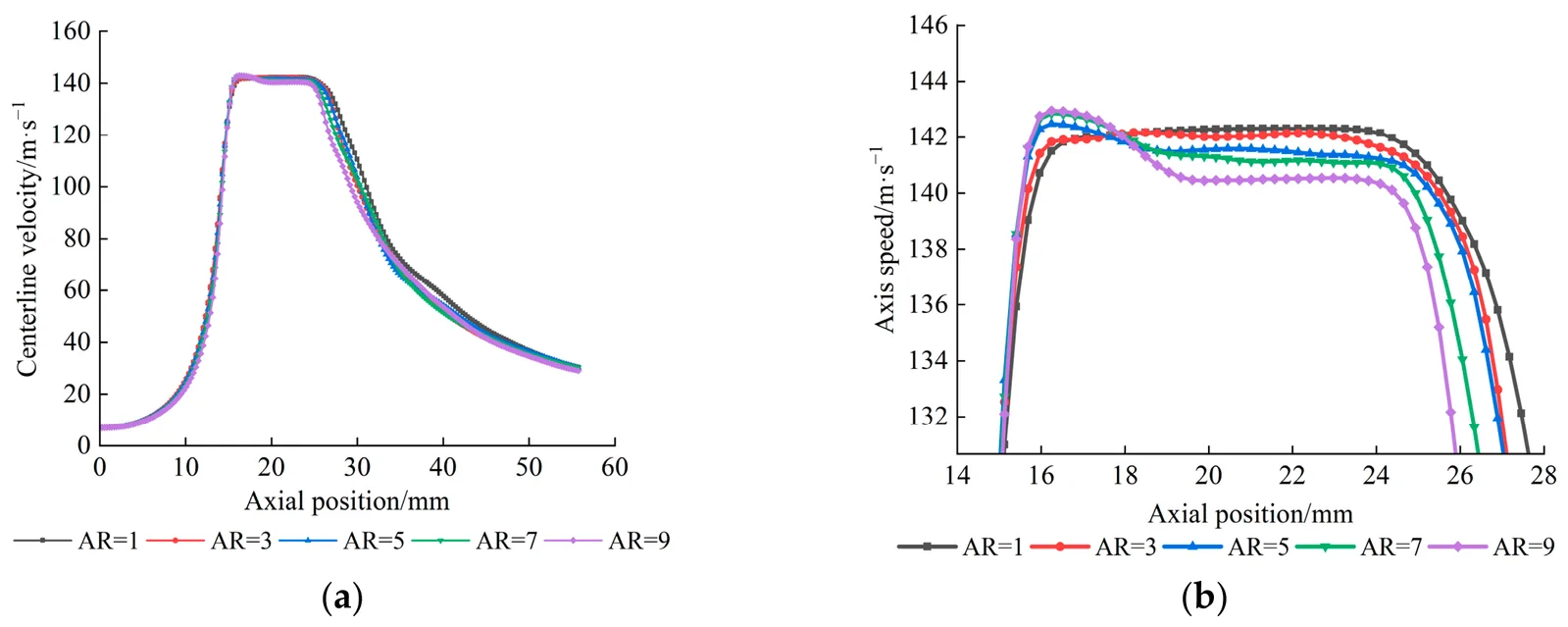
Variation in the time-averaged jet centerline velocity with axial distance for different aspect ratios. (**a**) Complete centerline velocity profile; (**b**) Enlarged view of the key region.

**Figure 7:**
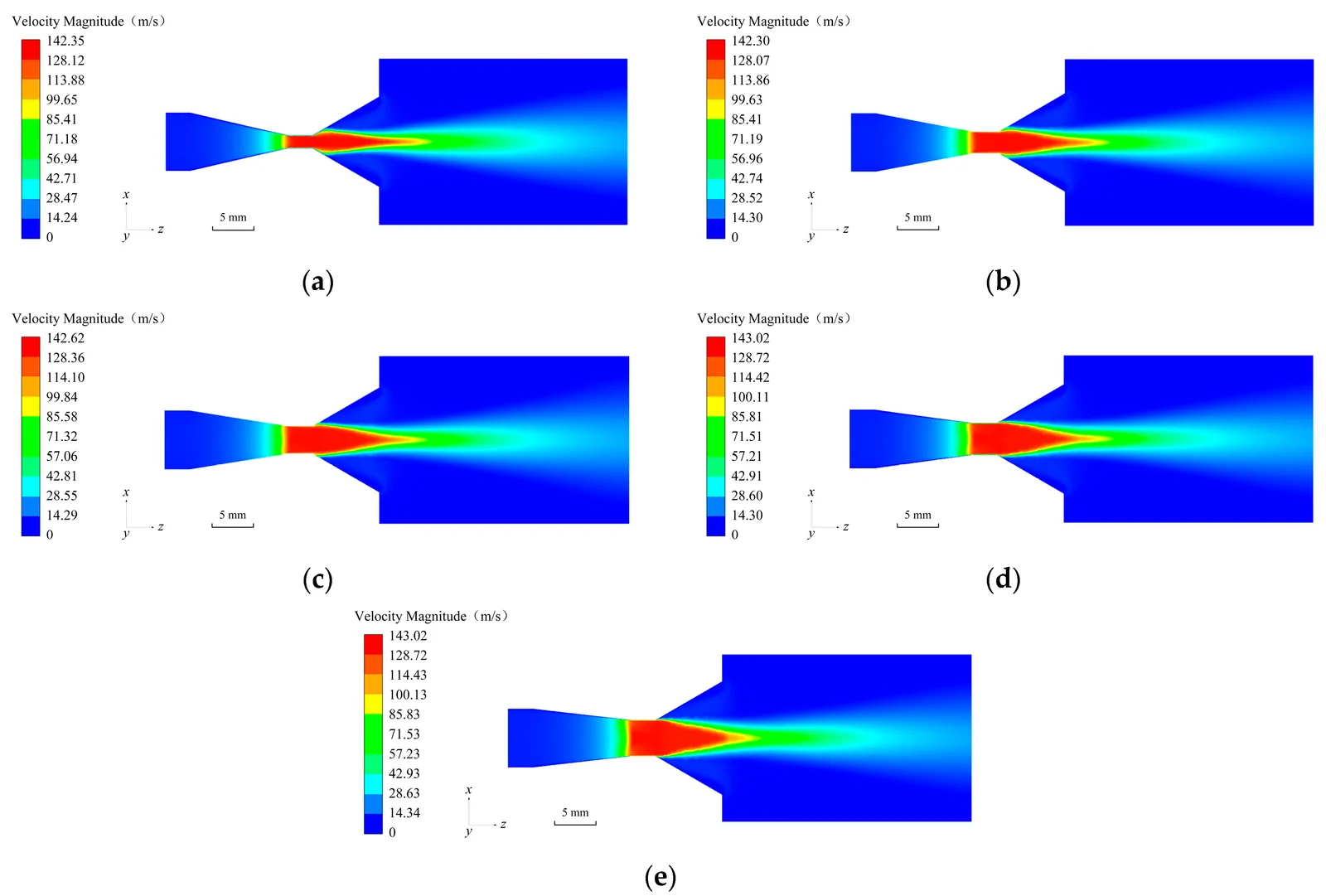
Time-averaged velocity distributions in the *y* = 0 plane. (**a**) AR = 1; (**b**) AR = 3; (**c**) AR = 5; (**d**) AR = 7; and (**e**) AR = 9.

**Figure 8:**
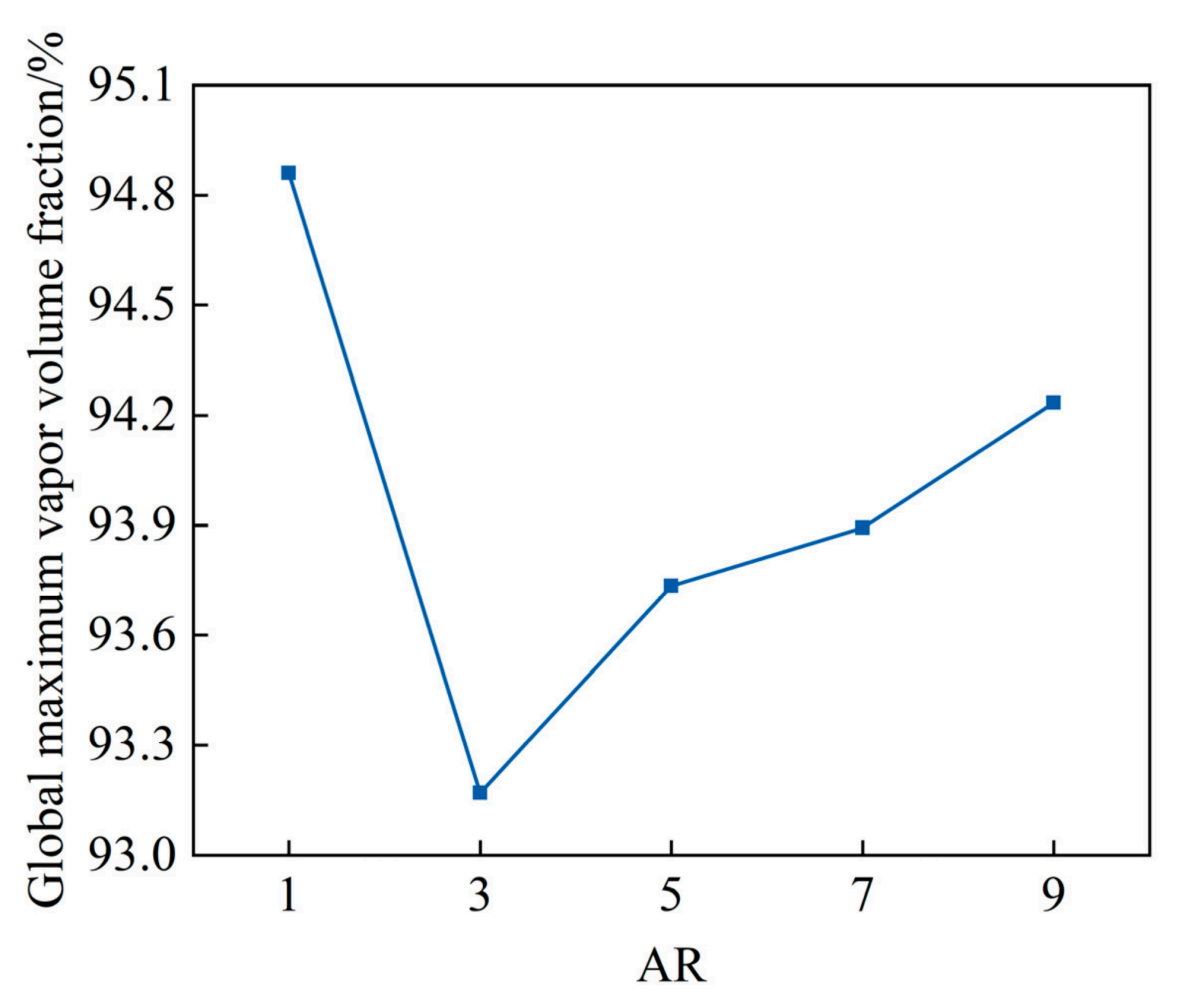
Global maximum of the time-averaged vapor-volume-fraction field for different aspect ratios.

**Figure 9:**
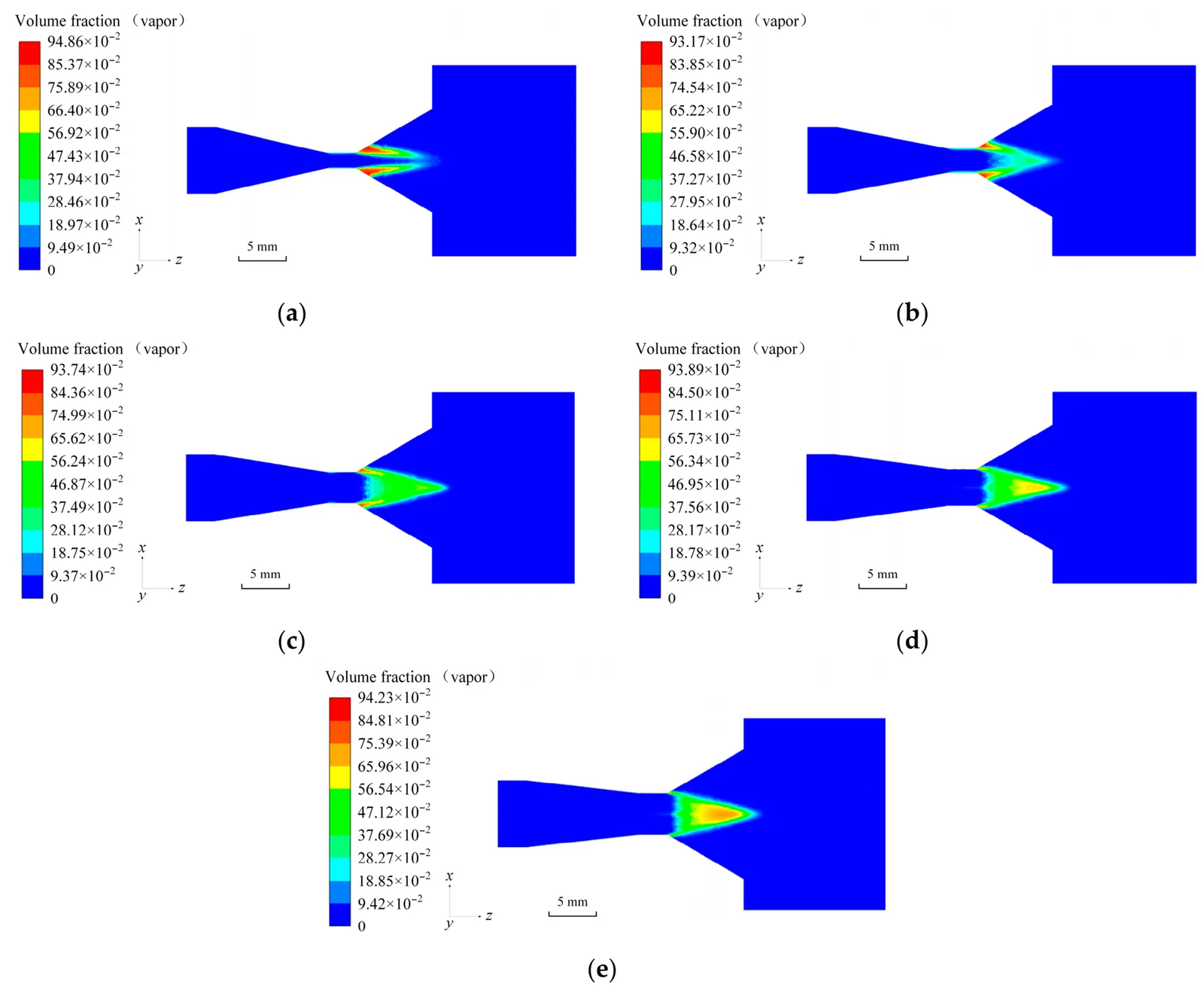
Time-averaged vapor-volume-fraction distributions in the *y* = 0 plane. (**a**) AR = 1; (**b**) AR = 3; (**c**) AR = 5; (**d**) AR = 7; and (**e**) AR = 9.

**Figure 10:**
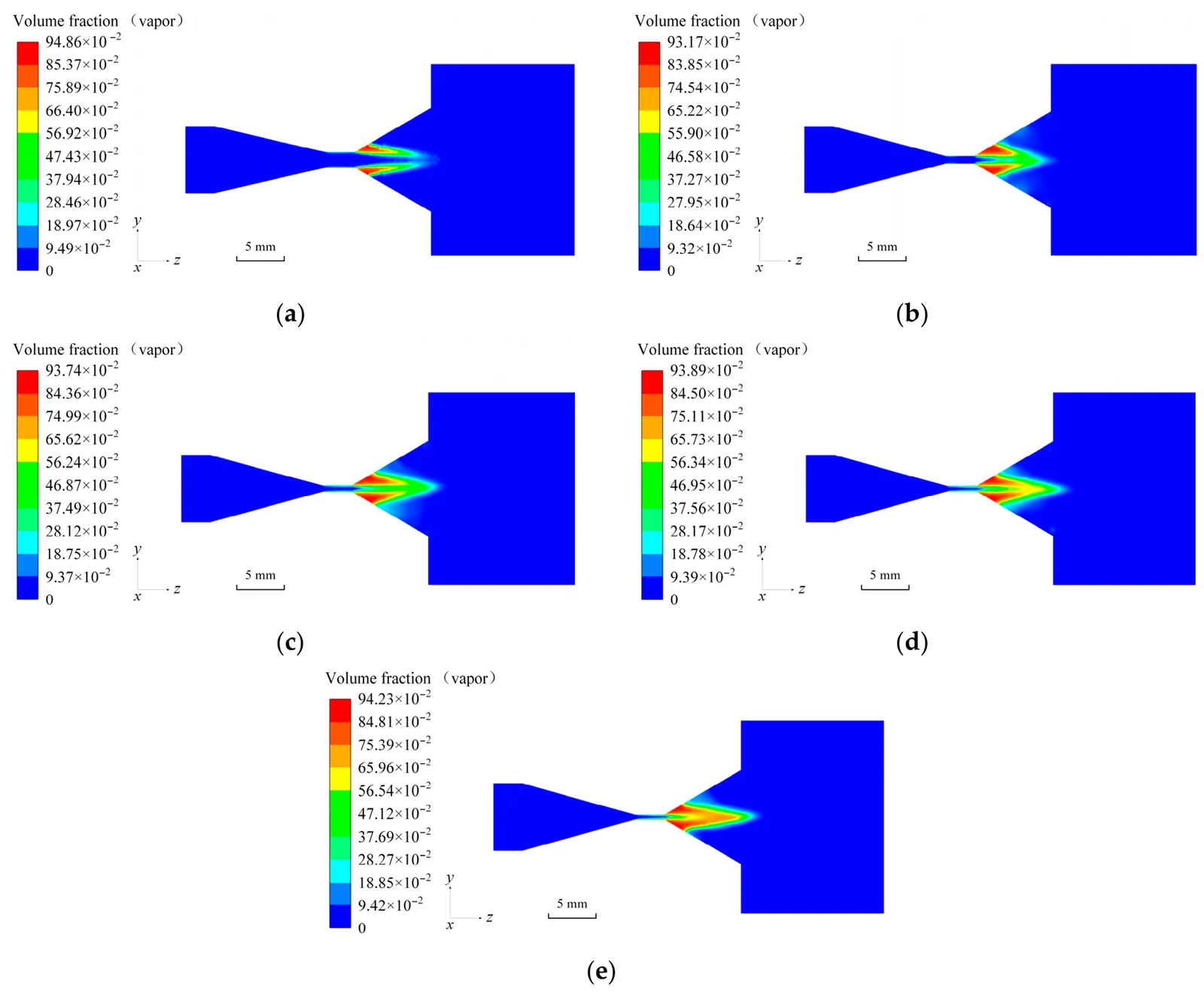
Time-averaged vapor-volume-fraction distributions in the *x* = 0 plane. (**a**) AR = 1; (**b**) AR = 3; (**c**) AR = 5; (**d**) AR = 7; and (**e**) AR = 9.

**Figure 11:**
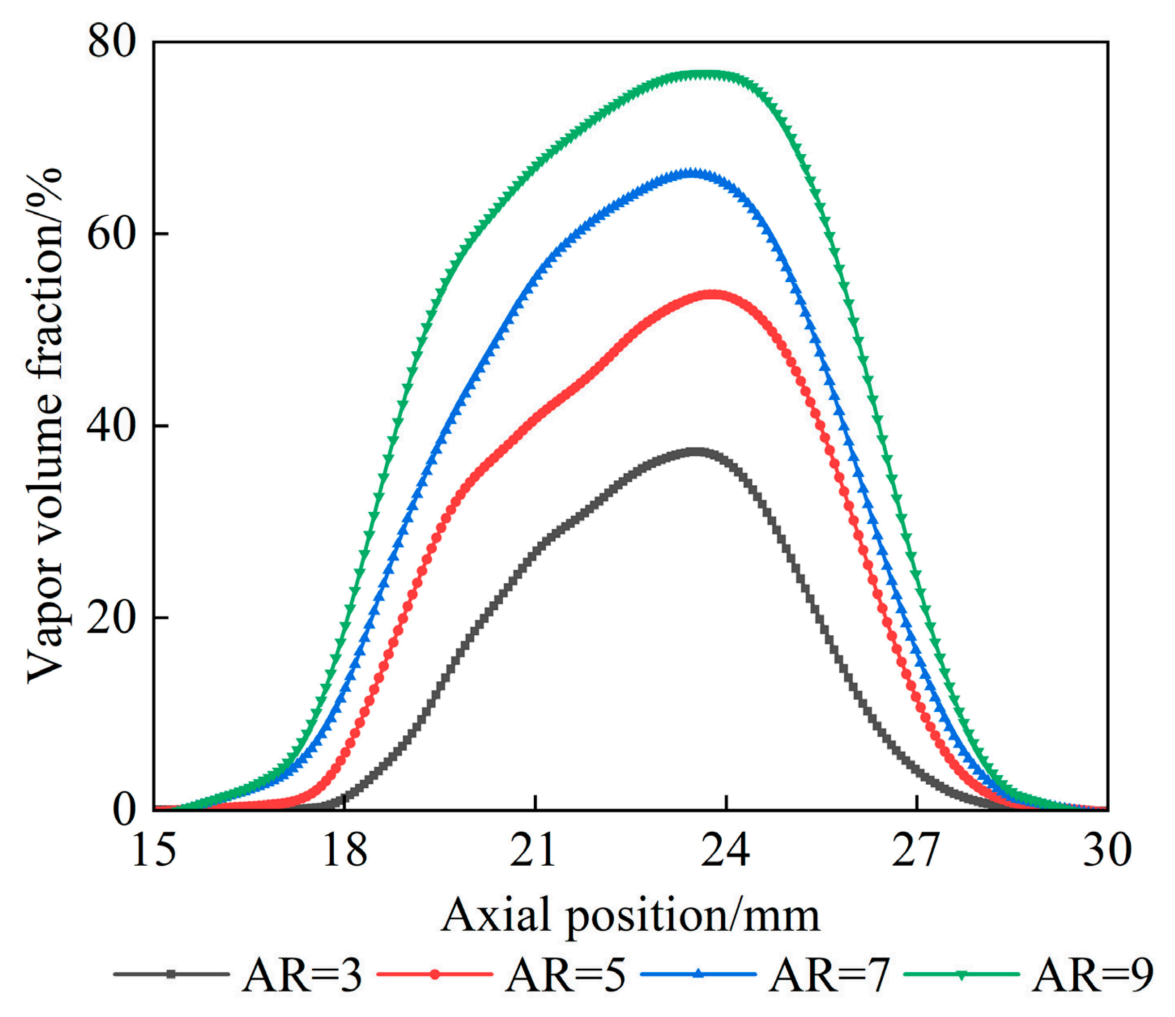
Axial distributions of the time-averaged vapor volume fraction along the jet centerline for the flat-slot nozzles.

**Figure 12:**
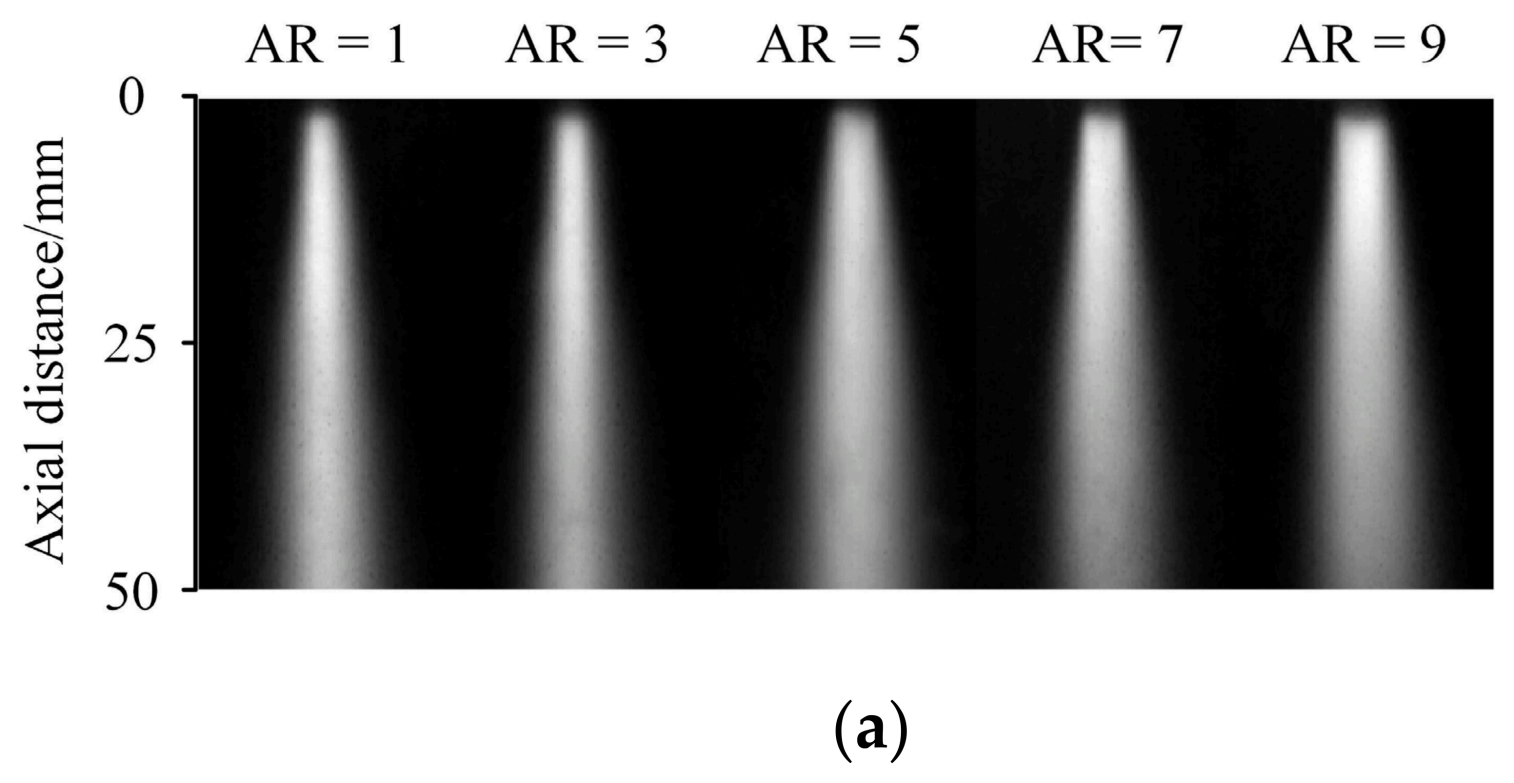

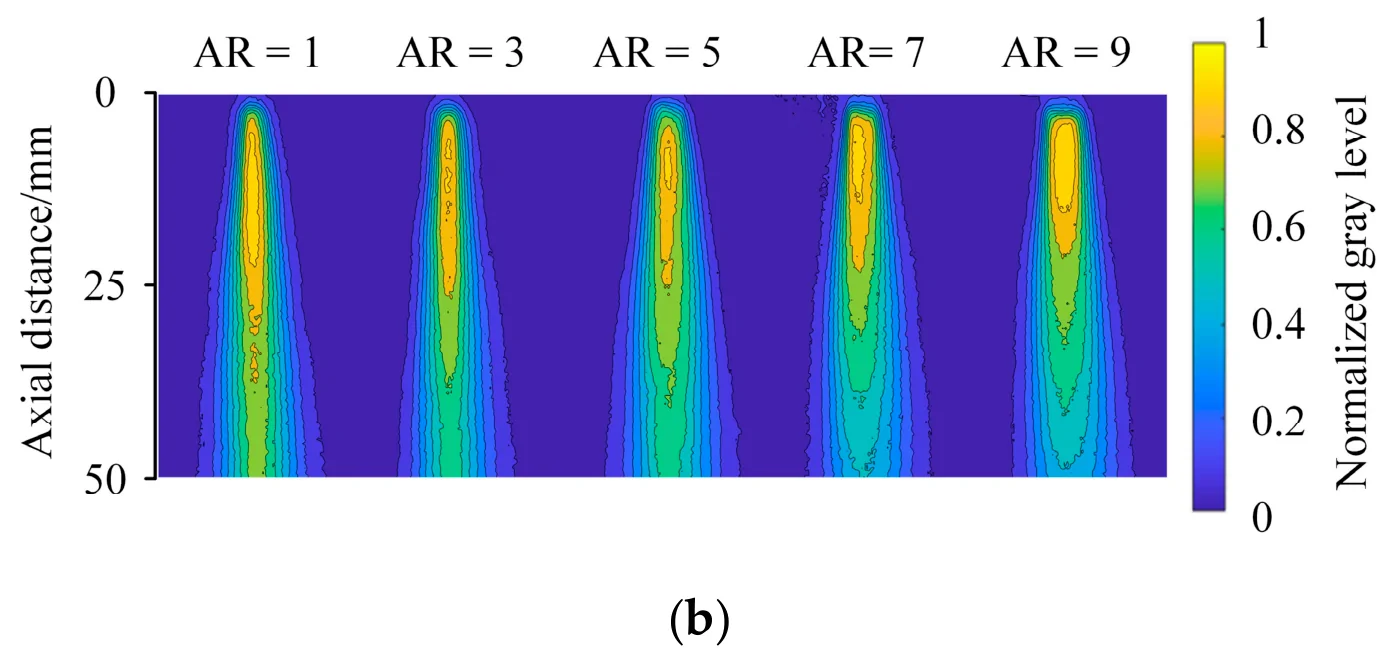
Time-averaged grayscale images and corresponding contour maps of the cavitating jets obtained from the high-speed imaging experiments. (**a**) Time-averaged grayscale images; (**b**) Time-averaged grayscale contour maps.

**Figure 13:**
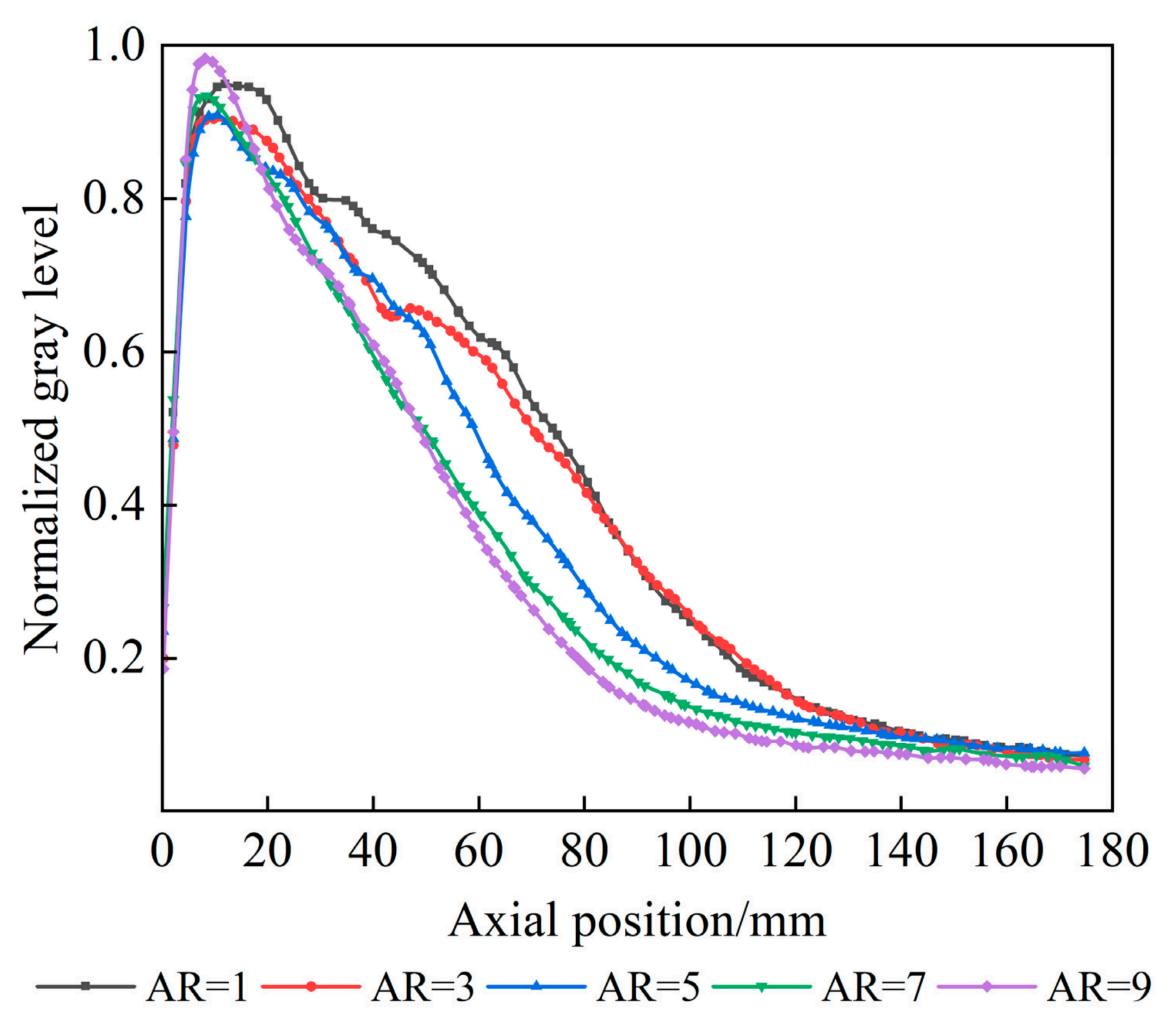
Axial distributions of the time-averaged gray level along the jet centerline extracted from the time-averaged high-speed imaging results.

**Table 1:** Geometric dimensions corresponding to different throat cross-sectional aspect ratios.

AR	Cross-Sectional Shape	Width *b* (mm)	Height *h* (mm)	Throat Cross-Sectional Area *A* (mm^2^)
1	Circular	1.4	1.4	1.539
3	Racetrack-shaped	2.230	0.743	1.539
5	Racetrack-shaped	2.836	0.567	1.539
7	Racetrack-shaped	3.334	0.476	1.539
9	Racetrack-shaped	3.767	0.419	1.539

**Table 2:** Actual geometric dimensions of the different nozzles.

Theoretical AR	Width *b* (mm)	Height *h* (mm)	Actual AR	AR Error (%)
1	1.392	1.374	1.013	1.31
3	2.259	0.744	3.036	1.21
5	2.803	0.562	4.988	0.25
7	3.365	0.477	7.055	0.78
9	3.743	0.419	8.933	0.74

**Table 3:** Mean wall *y*+ values for nozzles with different aspect ratios.

AR	Mean *y*+
1	1.14
3	1.46
5	1.28
7	2.02
9	2.30

**Table 4:** Results of the time-step-independence study.

Time-Step Size Δt (s)	Global Maximum of the Time-Averaged Vapor Volume Fraction (%)	Relative Difference (%)
5 × 10^−6^	94.529	0.262
2 × 10^−6^	94.389	0.113
1 × 10^−6^	94.234	0.051
5 × 10^−7^	94.354	0.076
2 × 10^−7^	94.282	Reference

**Table 5:** Geometric parameters of the high-gray-level regions in the time-averaged grayscale contour maps of the cavitating jets.

AR	Maximum Width *W_g_* (mm)	Maximum Length *L_g_* (mm)	Projected Area *S_g_* (mm^2^)
1	1.59	17.40	19.26
3	0.75	5.62	2.20
5	1.20	5.66	5.56
7	2.01	10.53	15.72
9	2.64	11.64	25.14

## Data Availability

The original contributions presented in this study are included in the article. Further inquiries can be directed to the corresponding author.
